# Hemin as a Molecular Probe for Nitric Oxide Detection in Physiological Solutions: Experimental and Theoretical Assessment

**DOI:** 10.1021/acs.analchem.4c01516

**Published:** 2024-05-03

**Authors:** Amir M. Alsharabasy, Pau Farràs, Abhay Pandit

**Affiliations:** † CÚRAM, SFI Research Centre for Medical Devices, University of Galway, Galway, Ireland H91 W2TY; ‡ School of Biological and Chemical Sciences, Ryan Institute, University of Galway, Galway, Ireland H91 TK33

## Abstract

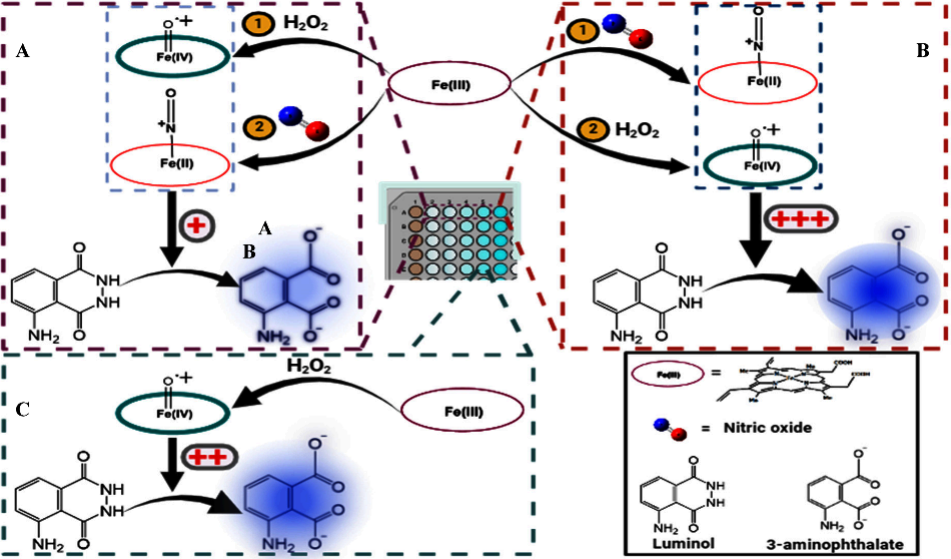

Given its pivotal role in modulating various pathological processes, precise measurement of nitric oxide (^●^NO) levels in physiological solutions is imperative. The key techniques include the ozone-based chemiluminescence (CL) reactions, amperometric ^●^NO sensing, and Griess assay, each with its advantages and drawbacks. In this study, a hemin/H_2_O_2_/luminol CL reaction was employed for accurately detecting ^●^NO in diverse solutions. We investigated how the luminescence kinetics was influenced by ^●^NO from two donors, nitrite and peroxynitrite, while also assessing the impact of culture medium components and reactive species quenchers. Furthermore, we experimentally and theoretically explored the mechanism of hemin oxidation responsible for the initiation of light generation. Although both hemin and ^●^NO enhanced the H_2_O_2_/luminol-based luminescence reactions with distinct kinetics, hemin’s interference with ^●^NO/peroxynitrite^–^ modulated their individual effects. Leveraging the propagated signal due to hemin, the ^●^NO levels in solution were estimated, observing parallel changes to those detected via amperometric detection in response to varying concentrations of the ^●^NO-donor. The examined reactions aid in comprehending the mechanism of ^●^NO/hemin/H_2_O_2_/luminol interactions and how these can be used for detecting ^●^NO in solution with minimal sample size demands. Moreover, the selectivity across different solutions can be improved by incorporating certain quenchers for reactive species into the reaction.

Nitric oxide (^●^NO) is a main gasotransmitter, responsible for the regulation of different biological activities, such as vasodilation,^1,2^ blood pressure,^3^ neuromodulation,^4^ and inflammation.^5^ Moreover, ^●^NO plays multiple roles in the progression of different types of cancers.^6−9^ Hence, different ^●^NO-releasing/generating compounds and materials have been used for modulating the levels of ^●^NO toward the treatment of different diseases as well as from basic research perspective.^10−13^ Thus, tremendous efforts were made toward the detection of ^●^NO in solutions, which rely mainly on chemiluminescence (CL)^14−16^ and amperometric detection.^17−19^ However, from the literature, two main sensitive techniques have been majorly employed for the real-time detection of ^●^NO in solutions, and these mainly rely on electrochemical and ozone-based CL methods.^20,21^ These two methods can detect minute concentrations of ^●^NO (in micromolar to picomolar) with a high resolution and tunable sensitivity and selectivity, particularly in the case of the amperometric detection of ^●^NO.^22^ Nonetheless, the foam produced because of nitrogen bubbling and the protein constituents within various culture media pose challenges to measurement sensitivity. Additionally, the need for substantial sample quantities and the associated high costs further hinder the widespread adoption of these techniques.^21^ Hence, there are still developmental stages for these techniques to overcome these drawbacks.

Hemin, the Fe(III)-protoporphyrin IX coordinate complex, has unique redox properties, responsible for its various catalytic functions.^23^ Hence, hemin has various applications for the detection of different analytes depending on its redox reactions, responsible mainly for either electrochemical oxidation–reduction reactions or luminescence generation. For instance, coordination polymer-hemin-based nanocomposites were employed for the detection of different biological analytes via amperometry and luminescence.^24^ Moreover, multiple G-quadruplex-hemin DNAzyme and RNAzyme systems with peroxidase-mimicking activity were developed for multidisciplinary applications.^25,26^ In addition, functionalization of graphene sheets with hemin was employed for the electro(chemiluminescence)-sensing of different biomolecules.^27,28^ Of interest, hemin-functionalized graphene through π–π stacking interactions produced a sensitive sensor for specific electrochemical detection of ^●^NO in physiological environments.^29^ Moreover, hemin was utilized as a main component in reaction systems for CL detection of certain biomolecules.^30−33^

Taking advantage of chemiluminescence that does not require light excitation for detection,^34^ tuneability of the assay to suit the composition of medium components, and the already reported hemin inducing effects for CL reactions,^35,36^ a hemin/luminol/H_2_O_2_-dependent system was employed for the detection of ^●^NO. We reported before the ability of hemin to scavenge ^●^NO and oxidize it into nitrite ions, besides hemin oxidation in the presence of H_2_O_2_.^37^ Here, the different interactions between hemin, ^●^NO, luminol, and H_2_O_2_ were investigated in different physiological solutions toward an understanding of the mechanisms leading to light generation. This was investigated experimentally and theoretically via density functional theory (DFT) calculations toward optimizing the conditions for detection of ^●^NO, released from two common ^●^NO-donors. Therefore, we present the fundamental principles of the proposed method for the continuous detection of ^●^NO using conventional CL reagents with minimal sample volumes as well as elucidate how specific agents may impact the luminescence kinetics. Understanding the reactions involved is essential for the development of an effective method for ^●^NO-detection, in terms of the use of a relatively small sample size, less sensitivity to the protein content of tested samples, and employing relatively inexpensive reagents.

## Experimental Section

### Chemiluminescence Measurements

The steps for the preparation of different solutions and tested compounds are in the Supporting Information. First, the effects of different reaction solutions, involving phosphate buffer (PB), fetal bovine serum (FBS)-free Dulbecco’s Modified Eagle Medium (DMEM), and FBS-containing DMEM (FBS/DMEM) in the presence and absence of hemin on the H_2_O_2_/luminol luminescence kinetics, were evaluated. Generally, the reagents were either added to every well of white opaque 96-well microplates containing different samples and mixed well or injected using the stopped-flow system in the Varioskan Flash microplate reader (Thermo Scientific, Finland). The measurement of luminescence intensity started instantly at two min intervals using the luminescence option in a microplate reader. The total volume of solution containing the different reaction components was 200 μL/well, and the concentration of the tested samples was modified accordingly. For recording the ultraviolet–visible (UV–vis) spectra, transparent 96-well microplates were used, and the absorbance was recorded with a microplate reader.

Second, the kinetics corresponding to ^●^NO released from different concentrations of (*Z*)-1-[*N*-(2-aminoethyl)-*N*-(2-ammonioethyl)amino]diazen-1-ium-1,2-diolate (DETA-NO) and sodium nitroprusside (SNP) in the presence and absence of hemin was analyzed in different solutions and was measured using UV–vis spectrophotometry as previously described. Third, the H_2_O_2_/luminol luminescence kinetics was measured following a mixture of DETA-NO, 3-Morpholinosydnonimine (SIN-1), hemin, and/or l-histidine (His). Third, the effects of CL reaction components on hemin-enhanced luminescence kinetics, including the concentration of H_2_O_2_, pH of buffer, and mixing method of all reactants, were investigated. For the pH effect, the CL reaction was performed in either phosphate (50 mM, pH 7.4) or carbonate buffer (50 mM, pH 10.5). Next, the influence of different concentrations of sodium nitrite (NaNO_2_) on the hemin-induced luminescence in PB was evaluated. Finally, for comparison, the effects of ferrous chloride (FeCl_2_), ferric chloride (FeCl_3_), and protoporphyrin IX (PPIX) on the main luminescence signal were studied.

### Computational Studies

The chemical structures of hemin (Fe(III)-Cl), hemin hydroxide (Fe(III)–OH), oxo iron(IV) porphyrin p-cation radical species **1** (Fe(IV)=O) and **2** (HO-Fe(IV)=O), and nitrosylated hemin [Fe(II)-NO]^+^ were drawn using ChemBioDraw Ultra 14 (PerkinElmer). The starting geometry for hemin was obtained from the crystal structure described before^38^ and then modified to get the structure of the other species. The quantum mechanics DFT calculations were carried out using the Gaussian 16 package,^39^ supported by the computational facilities of the Irish Centre for High-End Computing (ICHEC), as we described before,^40^ with details of calculations mentioned in the Supporting Information

## Results and Discussion

### Hemin Enhances the H_2_O_2_/Luminol-Based CL Reaction

**Figure 1:**
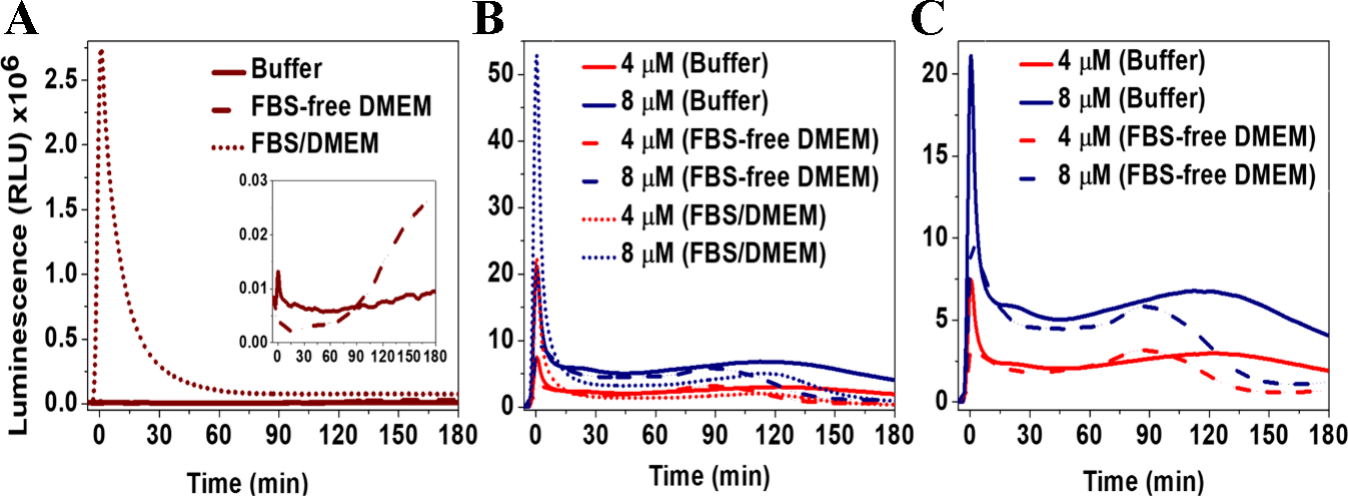
Luminescence kinetics, measured at 425 nm, following the addition of H_2_O_2_ and luminol in PB (50 mM, pH 7.4) to (A) hemin-free solutions composed of PB (solid wine curve), FBS-free DMEM **(dashed wine curve)**, and FBS/DMEM **(dotted wine curve)**. Inset: The luminescence curves are in the case of PB and FBS-free DMEM. (B) The kinetics curves in the case of 4 and 8 μM hemin-containing solutions, with (C) showing the differences between the kinetics in PB and FBS-free DMEM. Results are presented as mean luminescence intensity values, *n* = 3.

The injection of H_2_O_2_ and luminol enhanced the luminescence reading at different levels. The differences in luminescence kinetics depended on the composition of the reaction medium (Figure 1A). For instance, the luminescence intensity in PB was the lowest, with nearly constant values during the recording period. In the case of FBS-free DMEM, the luminescence increased after 90 min of reaction. However, a sharp increase in the intensity occurred once the reagents were injected into FBS-containing DMEM (FBS/DMEM). This flash luminescence was followed by glow kinetics, with nearly constant intensity over time after 90 min of reaction, but generally higher than that in the other solutions. To understand these changes, UV–vis spectra of luminol in PB and DMEM were obtained. Luminol has two characteristic peaks at 304 and 350 nm, attributed to the π → π* and π → π* transitions, respectively and their intensity decreased generally in the presence of H_2_O_2_ (Figure S1). This indicates an initiated oxidation of luminol, with the formation of luminol-monoanion, as an essential step toward the generation of light,^41,42^ as will be explained later. However, this decrease depended on the components of the reaction solution, with DMEM causing a sharper decrease in intensity compared to the buffer (Figure S1A,C). Moreover, the addition of FBS to both solutions enhanced this decrease in intensity and luminol oxidation (Figure S1B,D), which explains the observed differences in the CL kinetics. In the presence of hemin, a rapid increase in the luminescence intensity was detected, reaching its highest levels in FBS/DMEM, followed by buffer, with the lowest flash kinetics observed in FBS-free DMEM (Figure 1B,C). However, following the decay, the intensity values were nearly constant for 90 min in FBS/DMEM, with lower values than in other solutions, and the intensities in the case of the glow kinetics in PB were the highest. Moreover, in all solutions, the higher hemin concentrations caused a relatively higher luminescence compared to the lower ones, as explained in the next sections. Monitored by UV–vis spectroscopy, the inclusion of hemin with H_2_O_2_ in the luminescence reaction caused a more significant decrease in the absorption of luminol, which continued over time, compared to the hemin-free reactions (Figures 1A,B). This indicates the catalytic functions of hemin in this CL reaction. In general, the H_2_O_2_-induced luminol oxidation and hemin-enhanced luminescence intensity relate to a sequence of reactions shown in Table S1, summarized in Scheme S1, and discussed in the Supporting Information.

### The Interference of NO/Peroxynitrite (ONOO^–^) with Hemin Modulates Their Individual Effects on the H_2_O_2_/Luminol-Based CL Reaction

**Figure 2:**
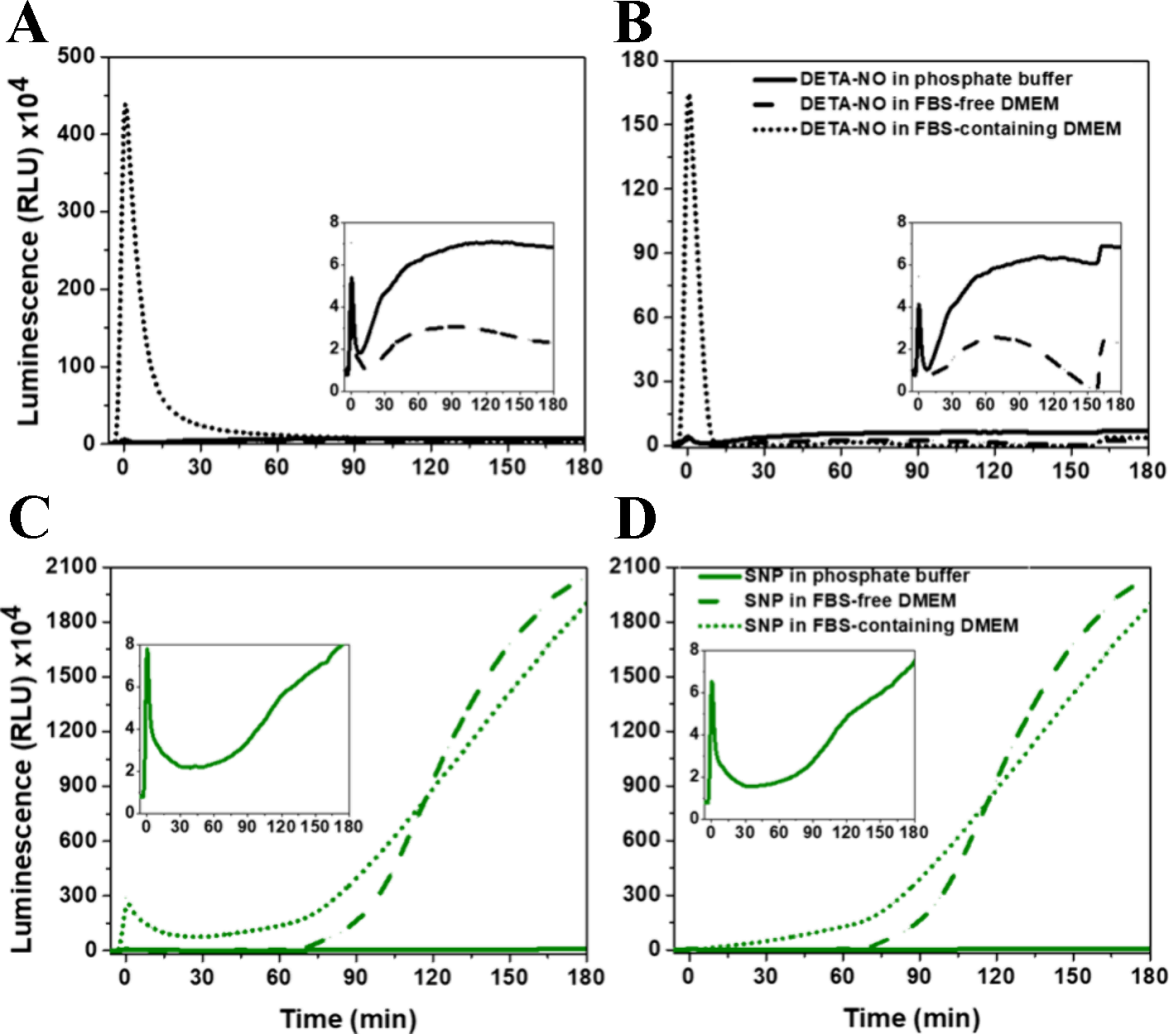
Luminescence kinetics, measured at 425 nm, following the addition of H_2_O_2_ and luminol in PB (50 mM, pH 7.4) to **(A,B)** 300 μM DETA-NO-containing PB (solid black curve), FBS-free DMEM (dashed black curve), and FBS/DMEM (dotted black curve), before and after subtraction of the luminescence values of the corresponding blanks, respectively. Insets: Luminescence kinetics was measured in PB- and FBS-free DMEM. **(C,D)** Kinetic curves for 1 mM SNP-containing PB (solid green curve), FBS-free DMEM (dashed green curve), and FBS/DMEM (dotted green curve) before and after subtraction of the luminescence values of the corresponding blanks, respectively. Insets: Luminescence kinetics was determined in the case of buffer. Results are presented as mean luminescence intensity values, *n* = 3.

Like the effects of hemin on the H_2_O_2_/luminol-luminescence kinetics, ^●^NO from different donors intensified the intensity. The ^●^NO-release kinetics from DETA-NO and SNP is different, which caused variances in the kinetics, and this also depended on the composition of the solution. In the case of DETA-NO, the released ^●^NO in FBS/DMEM caused a rapid increase in luminescence, followed by a fast rate of decay within the first 10 min of reaction, and, finally, a very slow rate of decrease in intensity (Figures 2A,B). In the other solutions, this initial increase in intensity was very weak, followed by a short-term luminescence decay, and then, after 15 min of reaction, the intensity started to increase over time, with generally higher levels in the buffer compared to both media. Using the electrochemical detection of ^●^NO, we previously reported its higher release rates from DETA-NO in PB than that in the FBS-containing medium.^37^ Moreover, as one of the nucleophilic/NO adducts, the decomposition of DETA-NO depends on the pH and temperature, with a higher stability in alkaline solutions and/or relatively lower temperatures.^43^ Hence, the induced luminescence in PB after 15 min correlates with the release of ^●^NO. However, the delayed sensing in the case of CL reactions relates to the different mechanisms of detection.

**Figure 3:**
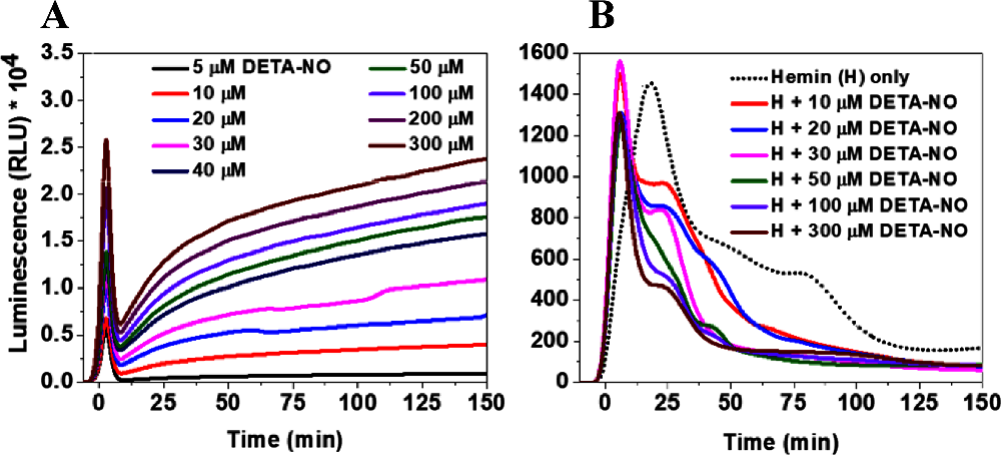
Luminescence kinetics, measured at 425 nm, following the addition of H_2_O_2_ and luminol in FBS-free DMEM to (A) different concentrations of DETA-NO only and (B) premixed solutions of hemin and DETA-NO with different concentrations in FBS-free DMEM. Results are presented as mean luminescence intensity values, *n* = 3.

Figure 3A compares the luminescence kinetics in DETA-NO/H_2_O_2_/luminol mixtures in FBS-free DMEM with different concentrations of DETA-NO. Once luminol and H_2_O_2_ were injected into the solution, and following the rapid amplification and decay of the signal, an increase in the luminescence intensity was detected, proportional to the ^●^NO-donor concentration. This luminescence is of glow-type, where the initial enhancement in luminescence was followed by an exponential increase in its intensity, which was more significant in the first 20 min of readings, particularly at DETA-NO concentrations higher than 10 μM. These amplified kinetics started to slow down then but without any decay within the recorded period. Of note, these results confirm our previously reported observations on the degradation of different DETA-NO concentrations and release of ^●^NO, measured electrochemically.^9^

However, in the presence of hemin, a change in luminescence kinetics was observed, where the light yield was relatively higher, and an intensive increase in luminescence started once the reaction was initiated. Here H_2_O_2_ and luminol were added to premixed hemin and DETA-NO for 10 min. The detected luminescence was of flash-type, which reached its maximum amplitude after 10 min, followed by decay, then a transition to intermediate glow-type luminescence before further decaying (Figure 3B). The rate of initial decay and the maximum intensity of the glow luminescence depended on DETA-NO concentration. While the transient amplitude was the highest in the case of 10 μM DETA-NO with hemin, with slight differences from 30 μM DETA-NO, the decay rate was the lowest. Moreover, it showed the longest period of glow luminescence with the highest intensity before further decay. Furthermore, the initial amplitudes during the flash kinetics due to different DETA-NO concentrations were less than that at 30 μM, with no significant differences between them. However, the decay rate reached its highest level at 300 μM DETA-NO, leading to the shortest and less intense glow luminescence, compared to the lower DETA-NO concentration. However, by the end of the recording period, the luminescence intensity of all DETA-NO/hemin mixtures reached the same level. These observations are explained in the next sections.

Using UV–vis spectroscopy, a sharp decrease in absorption was detected in mixtures of luminol, H_2_O_2_, and DETA-NO after 1 min of reaction but with slight changes observed after 15 min forward (Figure S2A). This indicated that the sharp initial ^●^NO release from DETA-NO is responsible for this initial high rate of oxidation, and as the release rate decreases over time, this oxidation rate decreases consequently. However, the inclusion of hemin with DETA-NO inhibited this decrease in absorbance after 1 min, indicating interactions between hemin and ^●^NO/ONOO^–^, which hinders the effects of each species on the CL reaction and luminol oxidation (Figure S2B). However, these effects were temporary, and the reaction was activated after that with enhanced oxidation of luminol at levels higher than those in the other groups. These effects were more significant after overnight incubation of the mixtures, where only the solution containing luminol, H_2_O_2_, hemin, and DETA-NO caused the sharpest drop in absorbance of luminol characteristic bands (Figure S2C). These latter results can be due to the aggregation of hemin molecules accompanied by partial deactivation of its inhibitory effects for luminol oxidation.

In the case of SNP, the luminescence kinetics followed a sigmoid function, which, in the case of both types of media, started with a low rate of CL reactions, causing a slight increase in the intensity (Figure 2C,D). This was followed by an exponential increase in intensity, which became significant after 15 and 75 min in FBS/DMEM and FBS-free DMEM, respectively. However, when SNP was dissolved in the buffer, the overtime enhanced luminescence was negligible compared to the kinetics in media. These effects refer to possible side reactions of culture medium components with SNP with a reducing power, causing enhanced SNP degradation and ^●^NO-release. SNP decomposition is sensitive to light and composition of the aqueous solution, which further controls the release of ^●^NO. In the presence of light, the bond between the central Fe(II) and NO^+^ ligand weakens, with a reduction of the later and subsequent release of ^●^NO.^44^ However, the presence of biological reductants is an essential factor for the metabolism and degradation of SNP. Various reducing agents were reported for inducing a one-electron transfer reduction of SNP and ^●^NO release, such as thiols^45^ and ascorbic acid.^46^

In addition, the decomposition of SNP in PB (pH 7.4) was negligible compared to that in the cysteine-containing solution, albumin solution, human plasma, and human blood, indicating a role for the components of these later solutions in the reduction of SNP.^47^ Hence, considering the inclusion of certain reducing agents and proteins (e.g., FBS) in the cell culture media can explain the observed enhanced luminescence due to SNP in the tested medium rather than that in the buffer. Moreover, the inclusion of FBS initiated an earlier CL reaction due to SNP compared with that in the FBS-free medium. Moreover, in contrast to the luminescence in the case of DETA-NO-containing solutions, the SNP-enhanced luminescence relates mainly to ^●^NO in addition to the side reactions of the produced Fe^2+^ ions with H_2_O_2_ through Fenton reactions.^45^ These ions are produced in the final stages of SNP decomposition in combination with ferrocyanide ions.

Deoxyhemoglobin is reported as a main reducing agent for SNP responsible for its bioactivation and release of ^●^NO.^48^ Via one-electron exchange reaction, methemoglobin, cyanide, and/or cyano-hemoglobin complex are formed alongside the released ^●^NO, which further nitrosylates the hemoglobin molecules.^49^ Moreover, we already reported how hemin increased the levels of ^●^NO, released from SNP, and the accumulation of nitrite in PB, suggesting a similar mechanism to that of hemoglobin, particularly after hemin nitrosylation.^37^ These findings explain the enhanced luminescence in the case of SNP/hemin-containing mixtures compared with SNP-only containing solutions.

### Hemin/NO-Induced CL Reactions

**Scheme 1:**
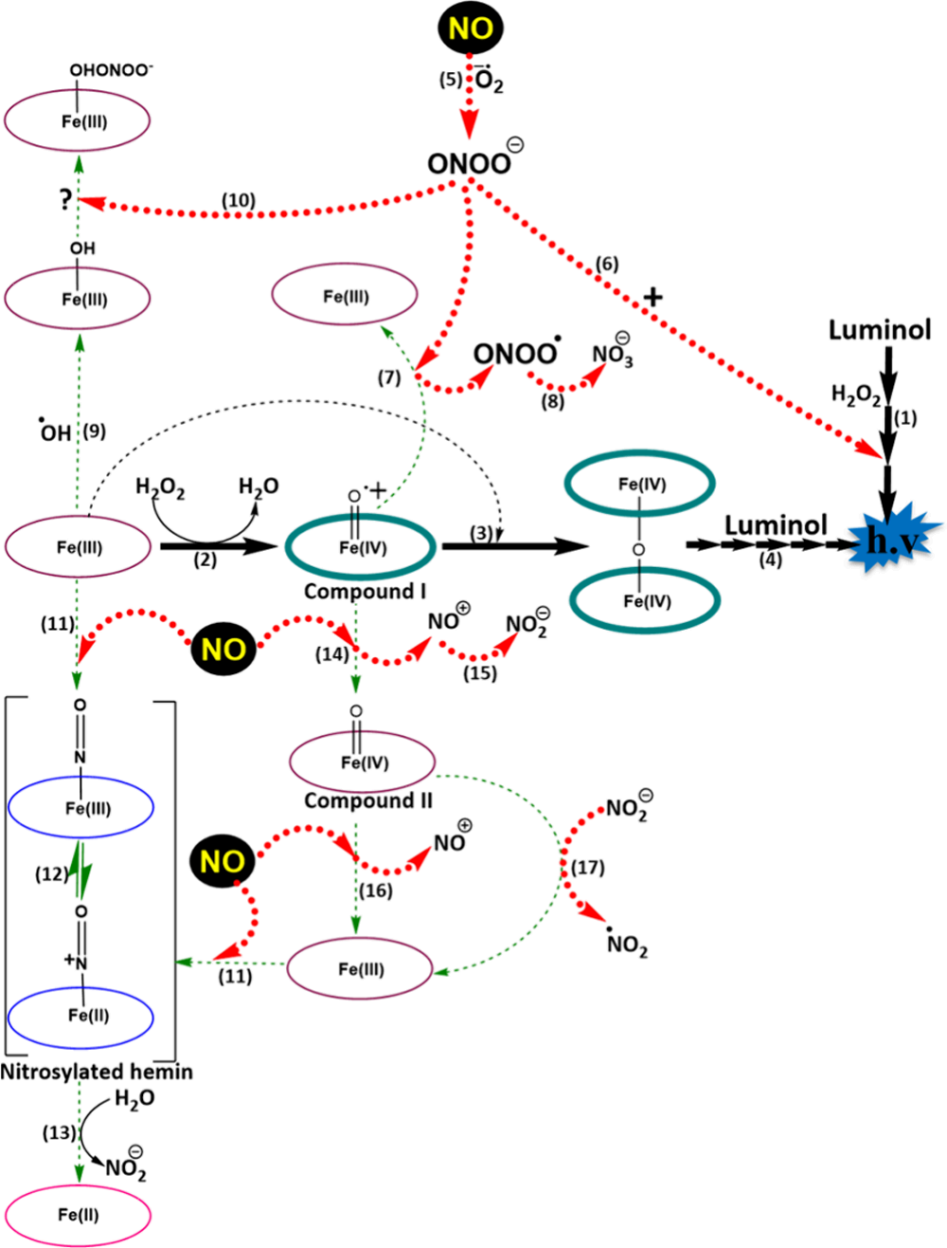
Reaction Mechanism Postulated for the CL Oxidation of Luminol in Alkaline Media in the Presence of ^●^NO and Hemin (Fe(III))

^●^NO is a reactive nitrogen species, which was proven to enhance the H_2_O_2_/luminol-based CL reaction.^50^ This is mainly due to the reaction between ^●^NO and the superoxide radical (O_2_^●–^), generated from H_2_O_2_, producing ONOO^–^ (Scheme 1, **Reaction 5**), which enhances the CL reaction, due to its strong oxidizing properties, relative to H_2_O_2_ itself (Scheme 1, **Reaction 6**).^51,52^ For instance, the rate constant for the reaction of the ONOO^–^ anion with sulfhydryls at pH 7.4 was reported to be 3 orders of magnitude greater than that of H_2_O_2_/sulfhydryls reactions.^51^ Moreover, following its protonation, the ONOO^–^ species were found to decompose into the strong oxidants: hydroxyl radicals (^●^OH) and nitrogen dioxide radical (^●^NO_2_),^53^ while its decomposition into nitroxyl anion (NO^–^) and singlet oxygen was reported as well.^54^ However, while the ^●^OH and ^●^NO_2_ species can also make the luminol oxidation thermodynamically feasible, their existence and the corresponding effects could not be confirmed when ONOO^–^ was formed in 20 mM PB at pH 7.4, referring to the particular role of ONOO^–^ as the luminescence enhancing species.^50^ This is also supported by the fact that the p*K*_a_ value for ONOO^–^ protonation is 6.6, so most of the formed species from the reaction of ^●^NO with H_2_O_2_ would exist as its conjugate base, ONOO^–^ in our buffer medium (50 mM PB, pH 7.4).

Scheme 1 and Table S2 summarize the CL reactions involving ^●^NO, ONOO^–^, and hemin, in addition to H_2_O_2_ and luminol as the main reactants. The presence of other radical species within the reaction medium or tested buffer can interfere with both the usual H_2_O_2_/luminol-based (Scheme 1, **Reaction 1**) and hemin/H_2_O_2_/luminol-based CL reactions (Scheme 1, **Reactions 2–4**), explained in detail in Table S1 and Scheme S1.

**Figure 4:**
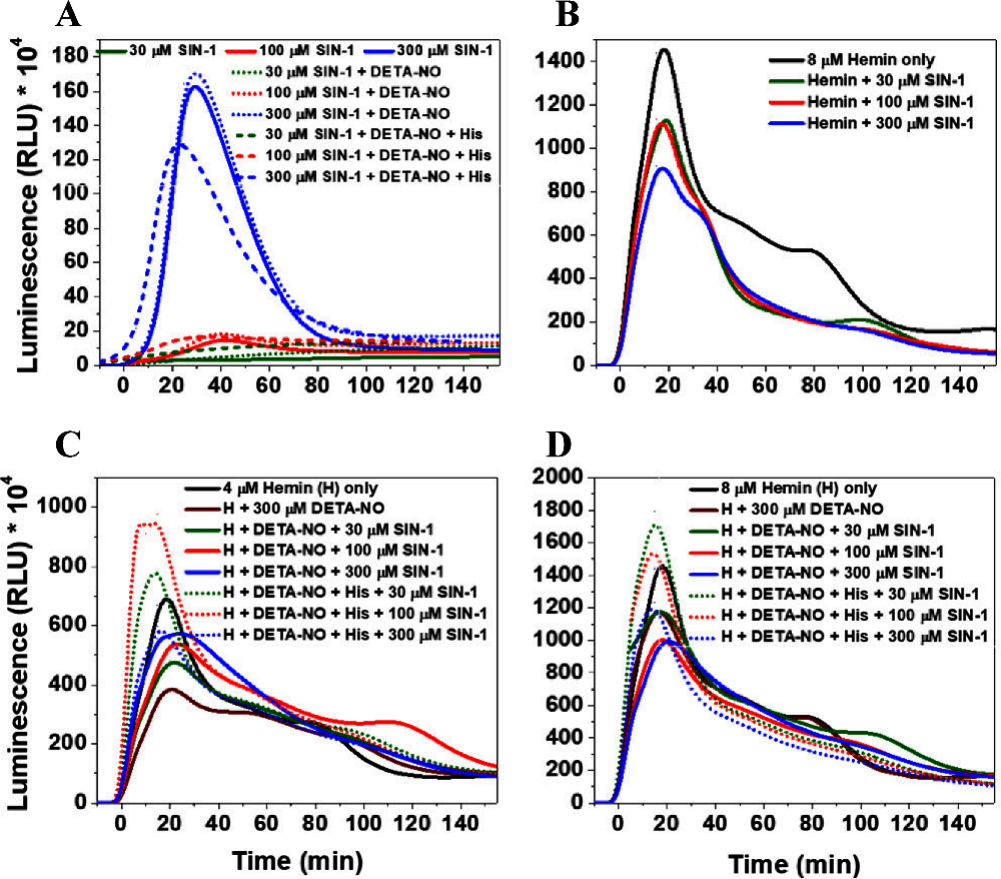
H_2_O_2_/luminol-based luminescence kinetics, measured at 425 nm, in response to (A) different concentrations of SIN-1 in the absence and presence of DETA-NO with and without His in phosphate buffer; (B) hemin mixed with different concentrations of SIN-1; and (C, D) hemin, at 4 and 8 μM, respectively, mixed with different concentrations of SIN-1 in the absence and presence of DETA-NO with and without His. The concentrations were as follows: H_2_O_2_, 50 mM; luminol, 1 mM; DETA-NO, 300 μM; His, 25 mM in PB (50 mM, pH 7.4). Results are presented as mean luminescence intensity values, *n* = 3.

**Figure 5:**
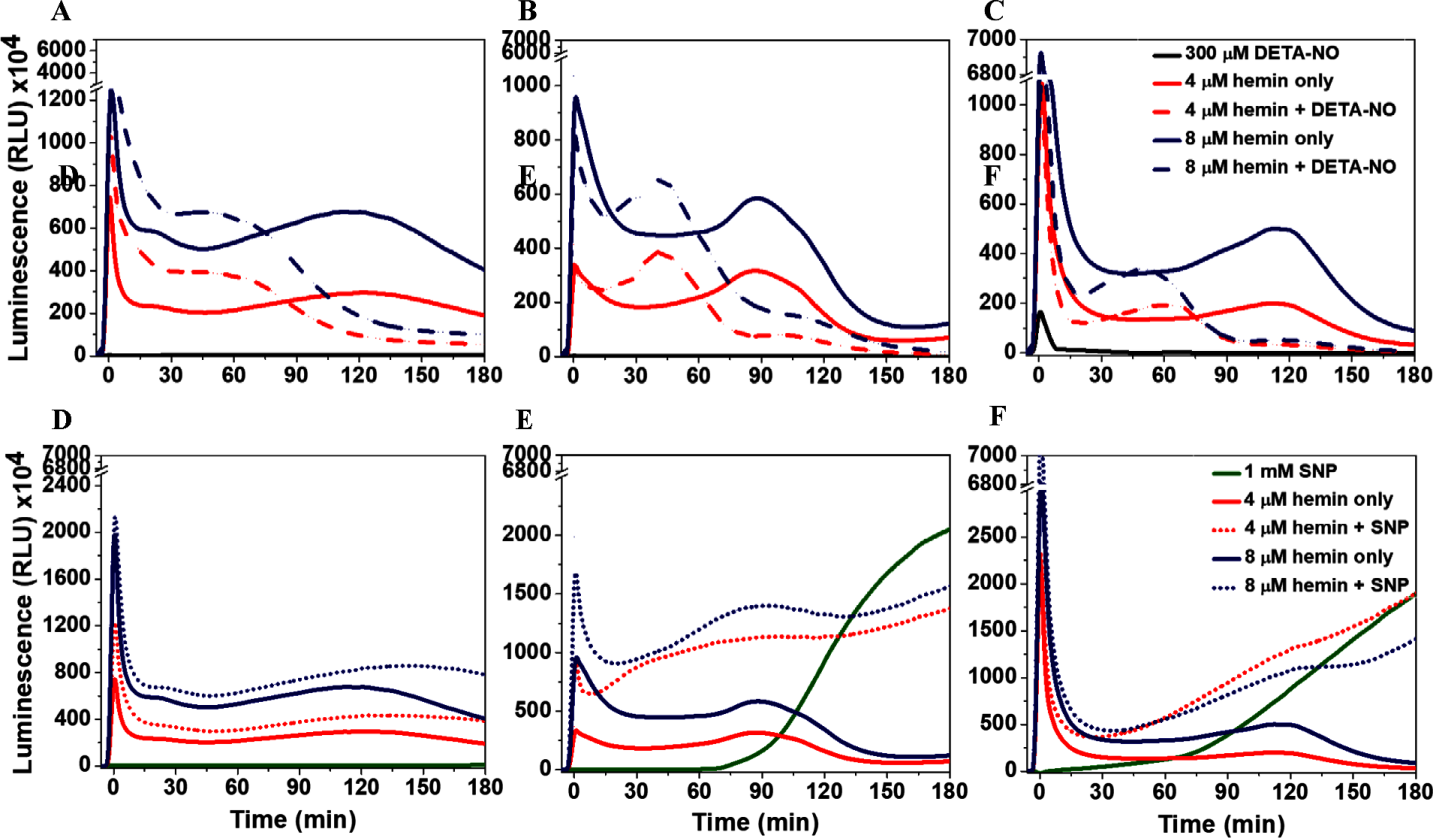
**(A-F)** The luminescence kinetics, measured at 425 nm, following the addition of 50 mM H_2_O_2_ and 1 mM luminol in PB (50 mM, pH 7.4) to PB (A), FBS-free DMEM (B), and FBS/DMEM solutions (C) containing 300 μM DETA-NO or different concentrations of hemin. **(D-F)** Kinetics due to the addition of PB (D), FBS-free DMEM (E), and FBS/DMEM solutions (F) containing 1 mM SNP and/or different concentrations of hemin. Results are presented as mean luminescence intensity values, *n* = 3.

SIN-1 decomposes by releasing the superoxide radical (O_2_^●–^), followed by generation of ^●^NO, which is rapidly scavenged by O_2_^●–^ forming ONOO^–^; hence, SIN-1 is considered a peroxynitrite donor.^55^ ONOO^–^ released from SIN-1 enhanced the luminescence signal under the current reaction setup, with a slight increase in intensity in the presence of DETA-NO (Figure 4A). However, the inclusion of His, reported as a quencher of ^●^OH and O_2_^●–^ species,^56,57^ in the reaction altered the detected luminescence, with a significant decrease in intensity at 300 μM SIN-1. These observations confirm the inclusion of these reactive species in the luminescence generation (Scheme 1, **Reactions 5, 6**), with concomitant enhancing effects of NO and ONOO^–^. In the presence of hemin, five main probabilities for the interference of ^●^NO with the hemin-induced CL reaction exist: **(1)** Reduction of compound I back to Fe(III), via the action of ONOO^–^ (Scheme 1, **Reaction 7**), which is further oxidized producing NO_3_^–^ ions (Scheme 1, **Reaction 8**).^58−60^**(2)** ONOO^–^scavenging, where hemin (Fe(III)) becomes hydroxylated forming hemin hydroxide (hematin monomer) (Scheme 1, **Reaction 9**), which further interacts with the ONOO^–^ ions, forming the Fe-[(OH)ONOO^–^]^2–^ complex (Scheme 1, **Reaction 10**).^61^ However, the exact mechanism of these reactions is not well-established. Moreover, although there is a possibility of hemin interaction with ONOO^–^, hemin did not cause any significant changes in the voltage readings, corresponding to the released ^●^NO and O_2_^●–^ species from SIN-1 (Figure S3A,B). This was the case whether SIN-1 was allowed to thaw for 10 and 30 min before injection into buffer, referring to a low probability of hemin-ONOO^–^ interactions. However, in the presence of SIN-1, an inhibition of hemin-induced CL reactions was detected, referring to the interactions between compound I and/or hematin with ONOO^–^ (Figure 4B). **(3)** Hemin nitrosylation following direct binding with ^●^NO (Scheme 1, **Reactions 11–13**),^37^ so the enhanced luminescence reactions due to ^●^NO (via ONOO^–^) and hemin (following its oxidation) are repressed. **(4)** Reduction of compound I to compound II via the action of ^●^NO (Scheme 1, **Reaction 14**), which is oxidized into nitrosonium (NO^+^) ions, and then to nitrite (NO_2_^–^) ions (Scheme 1, **Reaction 15**).^62,63^ Hence, **Reactions 11–15** (Scheme 1) explain the observed decreased luminescence intensity when hemin and DETA-NO are mixed with the luminescence reaction components (Figures 3B, 5C,D). For further understanding of these reactions, the hemin-induced luminescence kinetics was measured in the presence of His and DETA-NO. Although His is expected to cause quenching of the luminescence, it enhanced the intensity, and this was proportional to the hemin concentration (Figure S4A,B).

In different enzymatic systems, such as catalase and peroxidases, the protonated imidazole of His assists the two-electron oxidation of Fe(III) of the central hemin, with the formation of compounds I and II.^64^ This is responsible for the activity of the prosthetic group in these enzymes, and this can be the case within the studied CL reactions, responsible for the enhancement of hemin oxidation (Scheme S1, **Reaction 2**) and the subsequent light generation as observed. Furthermore, intermediate kinetics was detected when hemin, DETA-NO, and His were added to the CL reaction mixture, referring to a competition between hemin-NO binding and hemin oxidation. Moreover, it is noteworthy to mention that His, at the tested concentrations, did not significantly change the DETA-NO-induced luminescence. The native Fe(III)-protoporphyrin IX (PPIX) molecules can then be restored following the continuous reduction of compound-II in the presence of ^●^NO (Scheme 1, **Reaction 16**) or NO_2_^–^ ions (Scheme 1, **Reaction 17**). Like the effects of ^●^NO, the inclusion of NO_2_^–^ ions caused significant inhibition in the hemin-dependent luminescence, which was proportional to the concentration of NaNO_2_ (Figure S6).

To further understand the changes in electronic properties, particularly within the central iron following hemin oxidation and nitrosylation, DFT-based Quantum Mechanics calculations were performed, and the results were discussed in the Supporting Information, Tables S3–S7 and Figure S5. According to the experimental observations and theoretical calculations and due to the different competing reactions, two main sets of experiments were performed later: **(1)** Mixing of each tested compound with the CL reagents, followed by adding the ^●^NO-donor, and **(2)** Mixing of each tested compound with the ^●^NO-donor, followed by injection of the CL reagents.

### The CL Signal in Response to Hemin and Different NO-Donors

In the next set of experiments, hemin was diluted in the testing solution with or without mixing with ^●^NO-donor, followed by injection of the CL reagents. In all solutions, hemin enhanced an initial flash kinetics luminescence, and the intensity, after reaching its transient amplitude, started to decrease gradually, reaching a steady level after around 30 min of reaction (Figure 5). The maximum intensity during that phase depended on the components of the testing solution. It reached 920 × 10^4^ (2000 × 10^4^), 380 × 10^4^ (970 × 10^4^), and 2300 × 10^4^ (6900 × 10^4^) RLU in the case of 4 μM (8 μM) hemin in PB, FBS-free DMEM, and FBS/DMEM, respectively (Figure 5A-C). However, the second flash kinetics, following the glow luminescence, started after 60 min of reaction with a low light yield, and the maximum intensity was the same in all solutions for the same hemin concentration. However, this amplitude was reached after 90 min of reactions in FBS-free DMEM but after 120 min in the other solutions.

DETA-NO slightly enhanced the intensity of the transient amplitude in PB relative to hemin only, with no effects detected in FBS-free DMEM, but there was a significant decrease in FBS/DMEM. Following the decay, the second phase of flash kinetics was detected earlier than in the case of hemin only, with the same higher and lower transient amplitude in buffer, FBS-free DMEM, and FBS/DMEM, respectively. Taking into consideration the initial enhanced luminescence once the CL reagents were added to the DETA-NO solution in FBS/DMEM, which was higher than that in PB and FBS-free DMEM, the luminescence kinetics can be explained. These differences relate to either the FBS-promoting effects for the electron transfer and luminescence generation, as explained before, or to more generation of ONOO^–^ in FBS/DMEM compared with other solutions. However, considering the similar rates of ^●^NO released from DETA-NO, and so they produced ONOO^–^, in both FBS-free and FBS-containing solutions, the effects of FBS seem to be the main controlling factors for the enhanced luminescence in FBS/DMEM compared to those in FBS-free DMEM.

The increased luminescence in DETA-NO/hemin in the buffer may relate to the excessive production of ^●^NO compared to other solutions as we reported before,^37^ causing a reductive nitrosylation of hemin (Scheme 1, **Reactions 11–13)**, with further oxidation of Fe(II)-PPIX. This led to slightly enhanced luminescence generation in the case of DETA-NO in buffer. However, the lower rates of DETA-NO degradation in media with ^●^NO-release kinetics different than that in the buffer may accelerate ONOO^–^ generation once the CL reagents are added. Hence, the low amount of ^●^NO initially released, causing further nitrosylation of hemin, cannot compensate for the ONOO^–^-inhibiting effects of hemin-induced luminescence (Scheme 1, **Reactions 7 and 10**). This causes the ultimate decrease in the kinetics in FBS/DMEM.

The ^●^NO release rate from SNP in PB was the lowest among all solutions, and the kinetics due to the glow luminescence showed nearly equal intensities over the recording period (Figure 5D). However, in both media and corresponding to the continuous release of ^●^NO to the solution, the initial flash kinetics was followed by a short period of glow and then flash kinetics, and the luminescence intensities continued to increase over time (Figure 5E,F). The SNP/hemin mixtures enhanced the luminescence kinetics in all solutions compared to hemin only, and the kinetics, following the decay, was a result of the effects of both hemin and ^●^NO released from SNP. This enhancement generally relates to the fast release of ^●^NO from SNP, which ultimately activates the CL reactions as explained before for the DETA-NO/hemin in buffer. Moreover, we showed before that the addition of hemin to the SNP solution in PB caused an increased rate of ^●^NO release.^37^ Hence, this can be the same case here, causing an enhancement in the luminescence kinetics in SNP/hemin mixtures, as we explained before. However, as the luminescence in SNP/hemin showed lower intensity than SNP only starting after 130 min of reading, these relate to the interactions of ONOO^–^ with the Fe(III)-PPIX-related species, indicating a certain flux of ONOO^–^ required to react with hemin, which ultimately quenches the luminescence.

For comparison in another set of experiments, hemin was diluted in FBS-free DMEM, mixed with the CL reagents, and then SNP before the recording of the readings. Here, some changes in the luminescence kinetics were detected compared to those of the former injection cases. The transient amplitude was reached at 1100 × 10^4^ RLU after 11 min of reaction, followed by a slow rate of decay. In SNP/hemin mixtures, while the initial luminescence reading was similar to hemin only, the transient amplitude was reached at 7500 × 10^4^ RLU after 11 min, with a lower rate of decay, where the luminescence intensity increased after 25 min of reading, relative to hemin only (Figure S8). This general less luminescence in the presence of SNP refers to different reactions than in the case of injection due to the following possibilities: **(1)** the primary addition of CL reagents will initiate a series of reactions, involving the oxidation of the Fe(III)-PPIX structures toward the generation of light, and this reaction takes place in both tested sets of experiments. **(2)** When SNP is added next, the probability of Fe(III)-PPIX-NO binding will decrease (Scheme 1, **Reactions 11–13**) but with the same probability of its interactions with compounds I and II (Scheme 1, **Reactions 14 and 16**).

The latter reactions would be expected to decrease the rate of the CL reaction. However, as ^●^NO will convert to ONOO^–^ (Scheme 1, **Reaction 5**), this species will also help in the decrease of the hemin-induced CL reaction via its interference reactions (Scheme 1, **Reactions 7, 8**, and **10)**. The final species, produced from these reactions, will become a dead end for the Cl reaction in the absence of other reactants. However, as ^●^NO is continuously released over time with SNP degradation, a more prominent effect of ONOO^–^ alone can be observed in terms of enhanced luminescence after around 30 min of readings (Scheme 1, **Reaction 6**). Generally, although this mechanism will be expected in the case of the CL reagent injection, the prior mixing of SNP and hemin with the resulting Fe(III)-PPIX-NO binding will be expected to decrease the rate of hemin oxidation. Moreover, ^●^NO oxidation and conversion to ONOO^–^ become the prominent reaction responsible for the enhancement in the CL reaction rate combined with the reducing power of the hemin toward enhancing SNP.

## Conclusions

^●^NO functions as a signaling molecule, facilitating communication between adjacent cells and exerting regulatory effects on various biological redox processes. Here, the use of the hemin/luminol/H_2_O_2_-based CL reaction was proposed as a method for the instantaneous measurement of ^●^NO in diverse solutions. In the absence of hemin, ^●^NO showed inducing effects for the studied luminescence reactions, with the light intensity directly proportional to the concentration of the ^●^NO-donor. However, this intensity was generally low but significantly boosted in the presence of hemin. Furthermore, although the reaction rate of ^●^NO/ONOO^–^ with the reactive species involved in light generation was slower than that of hemin-induced luminol oxidation, the combination of ^●^NO with hemin enhanced the CL reaction. This underscores hemin’s role as both an enhancer of early light generation/detection and as a scavenger of ^●^NO, modulating the luminescence signal. Finally, the interaction mechanism between ^●^NO and its congeners, peroxynitrite and nitrite ions, with hemin-modulated luminescence was investigated. Hemin-modulated luminescence was investigated. In contrast to amperometric detection, CL detection requires a relatively small sample volume. We assessed and confirmed the potential interference of culture medium components on the CL chemiluminescence reaction and the resulting light. Therefore, this CL-based detection method presents a cost-effective means of sensing ^●^NO with comparable specificity to that of amperometric detection techniques. It can test up to 96 samples per run. Furthermore, employing freshly prepared reagents for each assay ensures sensitivity to minute levels of ^●^NO in solution, overcoming issues related to the electrode polarization time before each measurement. Based on these discoveries, ^●^NO/hemin/luminol/H_2_O_2_-based CL reactions represent a promising strategy, paving the way for the development of effective detection methods for ^●^NO in diverse solutions. In our laboratory, we are actively working on advancing the use of the studied reactions and addressing issues related to interference by other reactive species. Specifically, we are developing a novel ^●^NO-sensing platform utilizing hemin-decorated solid substrates for CL detection. This platform will undergo further exploration and refinement as part of our ongoing research.

## Supporting Information Available

The overtime change in the UV–vis spectra of luminol following mixing with H_2_O_2_; reaction mechanism postulated for the CL oxidation of luminol in the presence of hemin (Fe(III)); The overtime change in the UV–vis spectra of luminol following mixing with H_2_O_2_, DETA-NO, and hemin; the change in the UV–vis spectra of luminol with H_2_O_2_, hemin, DETA-NO; temporal changes in the voltage signal in response to SIN-1; the optimized molecular geometries of hemin hydroxide, nitrosylated hemin, iron(IV)-oxo species, and (HO-Fe(IV)=O)-containing species; the H_2_O_2_/luminol-based luminescence kinetics in response to hemin, DETA-NO, and l-histidine; the H_2_O_2_/luminol-based luminescence kinetics in response to hemin, NaNO_2_, mixtures of H_2_O_2_ and luminol, PPIX, FeCl_2_, or FeCl_3_; the luminescence kinetics in carbonate buffer in response to luminol/H_2_O_2_ mixture; the H_2_O_2_/luminol-based luminescence kinetics in response to SNP and hemin; reaction mechanism postulated for the CL oxidation of luminol in the presence of hemin; the main reactions involved in the hemin/H_2_O_2_/luminol-based CL reaction; the main reactions involved in the hemin/^●^NO/H_2_O_2_/luminol-based CL reaction; XYZ-files of optimized geometry of hemin hydroxide (FeC_34_H_33_N_4_O_5_), oxo iron(IV) porphyrin p-cation radical species 1 (Fe(IV)=O) ([FeC_34_H_32_N_4_O_5_]^+^), and oxo iron(IV) porphyrin p-cation radical species 2 (HO-Fe(IV)=O) ([FeC_34_H_33_N_4_O_6_]^+^); the optimized molecular geometries of hemin hydroxide, nitrosylated hemin, iron(IV)-oxo species, and (HO-Fe(IV)=O)-containing species; comparison of the distribution of the NPA charges among the optimized geometries of hemin, hemin hydroxide, oxo iron(IV) porphyrin p-cation radical species 1 and 2, and hemin in complexation with ^●^NO; the NEC in the iron atom of hemin (Fe(III)-Cl), hemin hydroxide, oxo iron(IV) porphyrin p-cation radical species 1 and 2, and hemin in complexation with ^●^NO (PDF)
