# Supplementary material for: Hemin as a Molecular Probe for Nitric Oxide Detection in Physiological Solutions: Experimental and Theoretical Assessment
Source: Anal Chem. 2024 May 3;96(19):7763–71. doi: 10.1021/acs.analchem.4c01516 (PMC11099896; doi:10.1021/acs.analchem.4c01516)
Supplement: Supplementary file 1 — ac4c01516_si_001.pdf [file ac4c01516_si_001.pdf]

# Supporting Information

# Hemin as a Molecular Probe for Nitric Oxide Detection in Physiological Solutions: Experimental and Theoretical Assessment

Amir M. Alsharabasy,[1*] Pau Farràs,[1,2] Abhay Pandit[1*]

[1]CÚRAM, SFI Research Centre for Medical Devices, University of Galway, Ireland. Postal code: H91 W2TY.
[2]School of Biological and Chemical Sciences, Ryan Institute, University of Galway, Ireland. Postal code: H91 TK33.

*AP: E-mail: Abhay.pandit@universityofgalway.ie
*AMA: E-mail: Amir.abdo@universityofgalway.ie

**TABLE OF CONTENT**

**List of schemes**

**Scheme S1.** Reaction mechanism postulated for the chemiluminescence (CL) oxidation of luminol in alkaline solution in the presence of hemin (Fe(III))……… ………………………. ……………………………….…………………………….........**S-7**

**List of table captions/legends**

**Supplementary Table S1.** The main reactions involved in the hemin/$H_2O_2$/luminol-based CL reaction and light generation……………………………………………………………………………………………………...............**S-6**

**Supplementary Table S2.** The main reactions involved in the hemin/$^{•}NO$/$H_2O_2$/luminol-based CL reaction and light generation.……………………………………………………………………….. ……………………….........................**S-10**

**Supplementary Table S3.** XYZ-files of optimized geometry of hemin hydroxide ($FeC_{34}H_{33}N_4O_5$) (B3LYP, GenECP, S = 3/2). The basis sets: LANL2DZ for Fe atom; 6-31+G(d,p) for H atom**,** 6-31+G(d,p) for C, O and N atoms................................................**S-12**

**Supplementary Table S4.** XYZ-files of optimized geometry of oxo iron(IV) porphyrin p-cation radical species **1** (Fe(IV)=O) ($[FeC_{34}H_{32}N_4O_5]^+$) (B3LYP, GenECP, S = 3/2). The basis sets: LANL2DZ for Fe atom; 6-31+G(d,p) for H atom**,** 6-31+G(d,p) for C, O and N atoms……………………………………………………...……………………………………..………..... **S-14**

**Supplementary Table S5.** XYZ-files of optimized geometry of oxo iron(IV) porphyrin p-cation radical species **2** (HO-Fe(IV)=O) ($[FeC_{34}H_{33}N_4O_6]^+$) (B3LYP, GenECP, S = 3/2). The basis sets: LANL2DZ for Fe atom; 6-31+G(d,p) for H atom**,** 6-31+G(d,p) for C, O and N atoms. ……………………………………………………………………….……………………………………...... **S-16**

**Supplementary Table S6.** Comparison of the distribution of the NPA charges among the optimized geometries of hemin (Fe(III)-Cl) (S = 3/2), hemin hydroxide (Fe(III)-OH) (S = 3/2), oxo iron(IV) porphyrin p-cation radical species 1 (Fe(IV)=O) (S = 3/2) and 2 (HO-Fe(IV)=O) (S = 3/2) and hemin in complexation with $^{•}NO$ based on the $[Fe(II)-NO]^+$ (S = 0) electronic state at the level of DFT/B3LYP/Gen ECP via Polarizable Continuum Model (PCM) using the integral equation formalism variant (IEFPCM). The

basis sets: LANL2DZ for the Fe atom, 6-31+G(d,p) for H atom, 6-31+G(d,p) for C, O, N and Cl atoms. All energies are in eV…… **S-18**

**Supplementary Table S7.** The NEC in the iron atom of hemin (Fe(III)-Cl) (S = 3/2), hemin hydroxide (Fe(III)-OH) (S = 3/2), oxo iron(IV) porphyrin p-cation radical species **1** ($[Fe(IV)=O]^+$) (S = 3/2) and **2** ($[HO\text{-}Fe(IV)=O]^+$) (S = 3/2) and hemin in complexation with •NO based on the $[Fe(II)\text{-}NO]^+$ (S = 0) electronic state with the occupation of the orbitals in the 3d level…… **S-19**

**List of figure captions/legends**

**Supplementary Figure S1.** The overtime change in the UV-Vis spectra of 200 µM luminol **(black curve)** following mixing with 10 µM $H_2O_2$ in FBS-free phosphate buffer **(A)**, FBS-containing phosphate buffer **(B)**, FBS-free DMEM **(C)** and FBS-containing DMEM **(D)** for one **(red curve)**, 30 **(blue curve),** 60 **(magenta curve),** 90 **(olive curve)** and 120 minutes **(orange curve).** Results are presented as mean absorbance values, n = 3…… **S-4**

**Supplementary Figure S2.** The overtime change in the UV-Vis spectra of 200 µM luminol **(black curve)** following mixing with 10 µM $H_2O_2$ and 60 µM DETA-NO **(A)** and with 10 µM $H_2O_2$, 1.6 µM hemin and 60 µM DETA-NO **(B)** for 1 **(red curve)**, 15 **(blue curve),** 30 **(magenta curve),** 45 **(olive curve)** and 60 minutes **(wine curve). (C)** The change in the UV-Vis spectra of 200 µM luminol **(black curve)** only and after mixing with 10 mM $H_2O_2$ only **(red curve)**, 10 mM $H_2O_2$, and 1.6 µM hemin **(blue curve),** 10 mM $H_2O_2$ and 60 µM DETA-NO **(magenta curve)**, and 10 mM $H_2O_2$, 1.6 µM hemin and 60 µM DETA-NO **(olive curve)** and overnight incubation. Results are presented as mean absorbance values, n = 3…… **S-8**

**Supplementary Figure S3.** The Temporal changes in the voltage signal in response to 1 mM SIN-1, prepared after thawing for 10 **(A)** and 30 min **(B)** and dissolving in phosphate buffer without **(black curve)** and with subsequent injection of 8 µM hemin **(blue curve)**. Results are presented as mean voltage values, n = 3…… **S-9**

**Supplementary Figure S4.** The optimized molecular geometries of hemin hydroxide **(A)**, nitrosylated hemin **(B)**, iron(IV)-oxo species **(C)** and (HO-Fe(IV)=O)-containing species **(D)**…… **S-11**

**Supplementary Figure S5.** The $H_2O_2$/luminol-based luminescence kinetics in response to 4 **(A)** and 8 µM hemin **(B)** in the absence and presence of DETA-NO with and without L-histidine (His) in phosphate buffer; The concentrations: $H_2O_2$: 50 mM; luminol: 1 mM; DETA-NO: 300 µM in phosphate buffer (50 mM, pH 7.4). Results are presented as mean luminescence intensity values, n = 3…… **S-18**

**Supplementary Figure S6.** The $H_2O_2$/luminol-based luminescence kinetics, measured at 425 nm, in response to 8 µM hemin mixed with different concentrations of $NaNO_2$. The concentrations: $H_2O_2$: 50 mM; luminol: 1 mM in phosphate buffer (50 mM, pH 7.4). Results are presented as mean luminescence intensity values, n = 3. …… **S-20**

**Supplementary Figure S7.** The luminescence kinetics in phosphate buffer (50 mM, pH 7.4), measured at 425 nm, in response to: **(A)** 1 mM luminol only, mixture of luminol and 50 mM $H_2O_2$ only, or hemin only, **(B)** mixture of 50 mM $H_2O_2$ and 1 mM fresh or one-week old luminol, **(C)** mixture of 1 mM fresh luminol and different concentrations of $H_2O_2$, **(D)** mixture of 1 mM luminol and 50 mM $H_2O_2$ in combination with different concentrations of protoporphyrin IX (PPIX), $FeCl_2$ or $FeCl_3$. **(E)** The luminescence kinetics in carbonate buffer (50 mM, pH 10.5) in response to a mixture of 1 mM fresh luminol and different concentrations of $H_2O_2$**.** Results are presented as mean luminescence intensity values, n = 3…… **S-22**

**Supplementary Figure S8.** The $H_2O_2$/luminol-based luminescence kinetics, measured at 425 nm, in response to SNP only, 8 µM hemin only, or after mixing of hemin with the CL reagents, followed by addition of SNP in phosphate buffer (50 mM, pH 7.4). Results are presented as mean luminescence intensity values, n = 3…… **S-23**

## EXPERIMENTAL SECTION

**Materials and Reagents.** Hemin, sodium phosphate dibasic dihydrate, ($Na_2HPO_4 \cdot 2H_2O$), sodium dihydrogen phosphate dihydrate ($NaH_2PO_4 \cdot 2H_2O$), sodium bicarbonate, sodium carbonate (anhydrous), sodium hydroxide (NaOH), hydrogen peroxide ($H_2O_2$, 30%), anhydrous dimethyl sulfoxide (DMSO), L-histidine (His), sodium nitrite ($NaNO_2$), ferrous chloride tetrahydrate ($FeCl_2$), ferric chloride ($FeCl_3$), Dulbecco's Modified Eagle Medium (DMEM), penicillin/streptomycin, L-glutamine, fetal bovine serum (FBS), and white opaque 96-well microplates were all purchased from Sigma-Aldrich. Luminol and transparent 96-well microplates were from Fisher Scientific. Protoporphyrin IX (PPIX) was supplied from Frontier Scientific. The following •NO donors were used during the study: sodium nitroprusside (SNP, Merck) and (Z)-1-[N-(2-aminoethyl)-N-(2-ammonioethyl)amino]diazen-1-ium-1,2-diolate (DETA-NO). 3-Morpholinosydnonimine hydrochloride (SIN-1) was supplied from Biotium.

**Preparation of reagents.** For the main experiments, the chemiluminescence (CL) reagents, luminol, and $H_2O_2$ were freshly prepared in phosphate buffer (PB) (50 mM, pH 7.4). Luminol solution was prepared by dissolving in DMSO: PB (1:4) solution and $H_2O_2$ was diluted from its 30% stock directly before the main experiment. The final concentrations of luminol and $H_2O_2$ were 1 and 50 mM, respectively, unless mentioned otherwise. Hemin was initially dissolved in DMSO, before fresh dilution in the respective testing solutions. PPIX solution was prepared freshly by dissolving in 0.1 M NaOH, followed by the addition of DMSO for a final 50/50 NaOH/DMSO solvent, then diluted in PB. $FeCl_2$, $FeCl_3$, $NaNO_2$, and His were dissolved freshly in the PB. The final concentrations of hemin, PPIX, $FeCl_2$, and $FeCl_3$ were 4 and 8 µM. A stock solution of DETA-NO was initially prepared by dissolving in 0.01 M NaOH, then freshly diluted in the tested solution just before the main experiment. SNP and SIN-1 were dissolved directly in the testing solution to prepare the finally tested concentrations.

**Computational studies**. The molecular geometries of all structures were first optimized *in vacuo* using the B3LYP hybrid functional with the 6-31+G(d,p) basis set on all atoms**,** followed by another *in vacuo* optimization stage, where Fe was described with the LANL2DZ basis set. Here, C, O, Cl, and N atoms were described by the 6-311+G(d,p) basis set and H atoms by the 6-31+G(d,p) basis set. After reaching its minima, the -Cl in hemin structure was exchanged with -OH forming Fe(III)-OH (S = 3/2), =O forming oxo iron(IV) porphyrin p-cation radical species (Fe(IV)=O) (S = 3/2) or (HO-Fe(IV)=O) (S = 3/2), or with -NO forming $[Fe(II)\text{-}NO]^+$ (S = 0), with repeating of the optimization and frequency calculations at the assigned multiplicities. Next, the single point energy (SPE) calculations were performed at the Becke, 3-parameter, Lee–Yang–Parr (B3LYP/cc-PVTZ) levels both *in vacuo* and in water using the integral equation formalism variant (IEFPCM)**.** Next, the natural atomic charges in all compounds were computed at the level of B3LYP/cc-PVTZ using the Gaussian NBO Version 3.1, with the calculation of the frontier molecular orbitals.

RESULTS

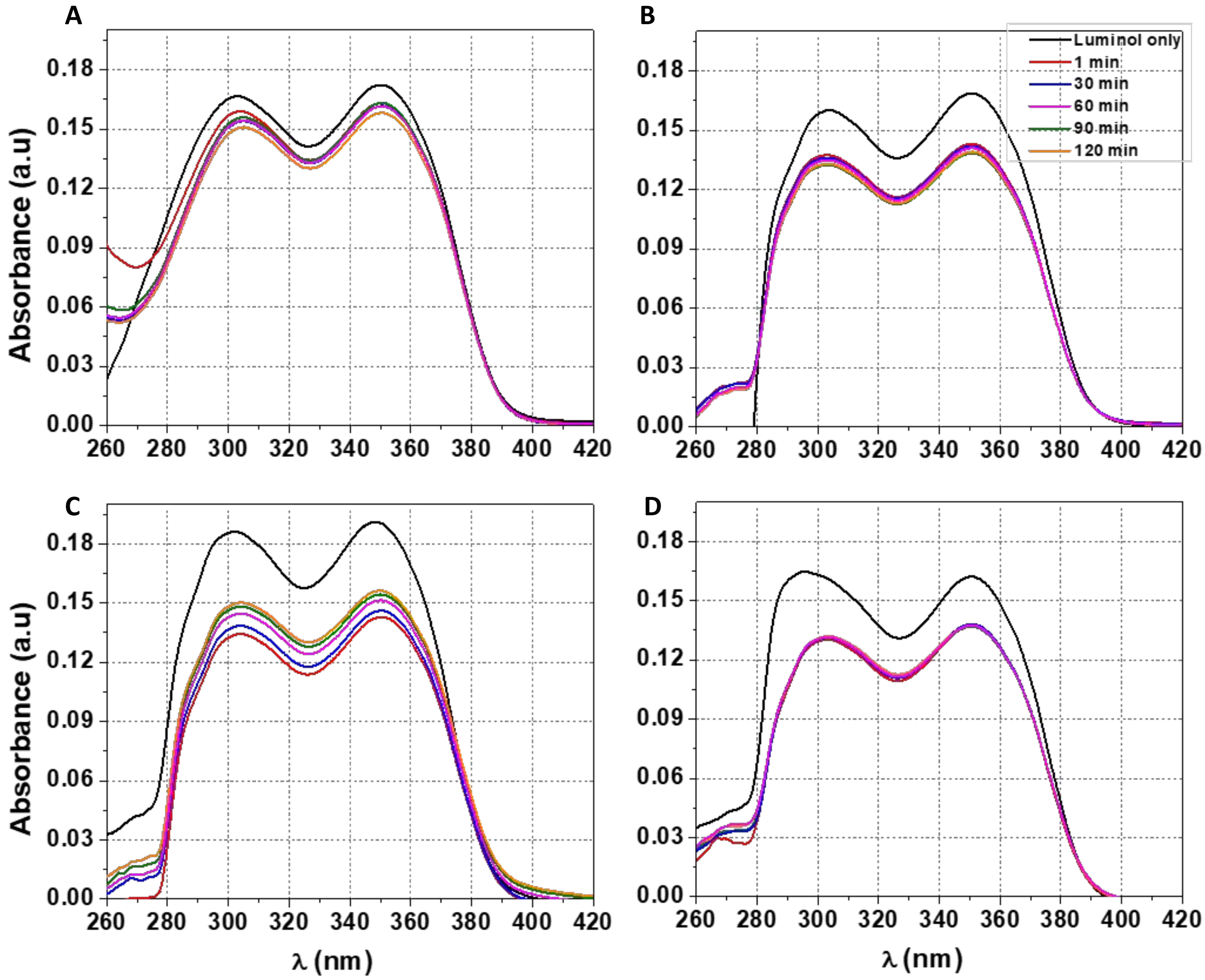


**Figure S1.** The overtime change in the UV-Vis spectra of 200 μM luminol **(black curve)** following mixing with 10 μM $H_2O_2$ in FBS-free phosphate buffer **(A)**, FBS-containing phosphate buffer **(B)**, FBS-free DMEM **(C)** and FBS-containing DMEM **(D)** for one **(red curve)**, 30 **(blue curve),** 60 **(magenta curve),** 90 **(olive curve)** and 120 minutes **(orange curve).** Results are presented as mean absorbance values, n = 3.

**Hemin enhances the $H_2O_2$/luminol-based CL reaction.**
**(Supporting Data)**

**Table S1** and **Scheme S1** summarize the sequence of reactions involved in the $H_2O_2$-induced luminol oxidation and hemin-enhanced luminescence intensity.

**Reactions 1** and **2:** $H_2O_2$-induced oxidation of hemin (Fe(III)) forming compound I in the form of Fe(IV)=O (ferryl) center, combined with a porphyrin π-cation radical.[1,2] The rate constant for these oxidation reactions in the case of horseradish peroxidase (HRP) is in the order of $10^7$ $M^{-1}$ $s^{-1}$.[3,4]

**Reaction 3:** compound I species are quickly reduced by excess unoxidized hemin molecules (Fe(III)) forming μ-oxo bridged Fe(IV) dimer.

**Reaction 4:** oxidation of luminol forming a luminol mono and dianions, and this is considered the rate-limiting step for the luminescence reaction under normal conditions.[5] The pKa for this hydrogen subtraction is 6.3 and 12 to form mono and dianions, respectively, so at pH 9, about 98% of luminol would exist in its monoanion form.[6-8]

**Reaction 5:** the monoanion is then oxidized into a luminol anion in the presence of the Fe(IV) dimer, a molecular species with a high oxidation power, and forms a dimer composed of μ-oxo bridged Fe(IV) and Fe(III) monomers. This reaction is slower than **Reaction 4**, with a rate constant of 2.3 x $10^4$ $M^{-1}$ $s^{-1}$ in the case of HRP.[9,10]

**Reaction 6:** the formed dimer catalyzes also the further generation of oxidized luminol species from luminol anion, with the formation of μ-oxo bridged Fe(III) dimer.

**Reaction 7:** Disproportionation of each two anion molecular species into luminol and its diazaquinone derivative (indicated by a red circle), with a rate constant of 5 x $10^8$ $M^{-1}$ $s^{-1}$.[11]

**Reactions 8** and **9:** In the presence of hydroperoxide anion ($HOO^-$), the later species form the excited singlet 3-aminophthalate (3-AP*), with a rate constant of 5 x $10^7$ $M^{-1}$ $s^{-1}$.[12] The rates of these reactions are proportional to the $H_2O_2$ concentration, pH of solution, and pKa for dissociation of the luminol-peroxide intermediate into 3-AP* is within the range: 11.1-12.1.[7,13]

**Reaction 10:** fluorescence light is finally generated following the radioactive decay of this excited species. The **Reactions 8-10** were reported to be very fast.[5,14] Accordingly, the enhancement in the measured luminescence intensity due to $H_2O_2$/luminol reaction in the presence of hemin can be employed as an indication of the concentration and oxidation activity of hemin in solution. Moreover, for these reactions to continuously proceed, the formation of μ-oxo bridged Fe(IV) dimer is essential, for catalyzing the generation of peroxidatically active intermediates, and this depends on both hemin concentration and pH of the solution.[15] However, other side reactions take place once hemin is diluted in the aqueous solution, involving the dimerization of its molecules through the formation of μ-oxo bridge between each two molecules or via stacking of two or more molecules **(Reaction 11).**[16] This alters the local electronic structure at the central iron, inhibits its catalytic effects, and produces less light.[17] The effects of FBS/DMEM on the initial luminescence signal may be due to the interactions of FBS or one of its components with the oxidation of luminol and transferring of electrons between the excited species. A similar behavior was reported in the case of BSA, which was suggested to accelerate the electron transfer rate of 3-aminophthalate, responsible for its promoting effects for the CL intensity.[18]

**Table S1.** The main reactions involved in the hemin/$H_2O_2$/luminol-based CL reaction and light generation.

<table>
<tr><th>NO.</th><th>REACTION</th><th>REFERENCES</th></tr>
<tr><td>1</td><td>Fe(III)-Cl → $Fe(III)^+ + Cl^-$</td><td rowspan="3">2-4</td></tr>
<tr><td>2</td><td>$Fe(III)^+ + H_2O_2$ → $Fe(IV)=O^{\bullet +} + H_2O$</td></tr>
<tr><td>3</td><td>$Fe(IV)=O^{\bullet +} + Fe(III)^+$ → Fe(IV)-O- Fe(IV)</td></tr>
<tr><td>4</td><td>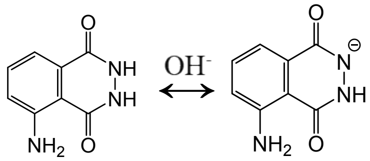
</td><td>5-8</td></tr>
<tr><td>5</td><td>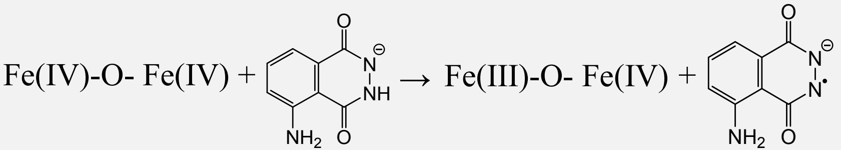
</td><td>9,10</td></tr>
<tr><td>6</td><td>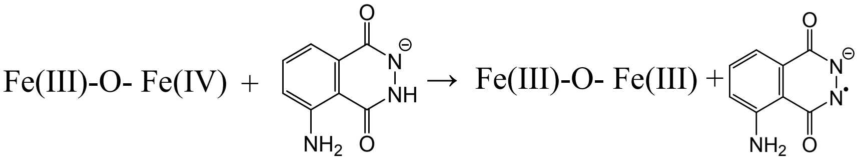
</td><td></td></tr>
<tr><td>7</td><td>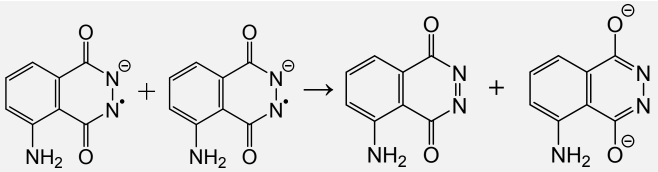</td><td>11</td></tr>
<tr><td>8</td><td>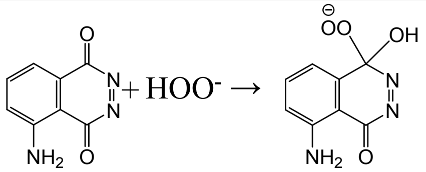
</td><td rowspan="2">12,13</td></tr>
<tr><td>9</td><td>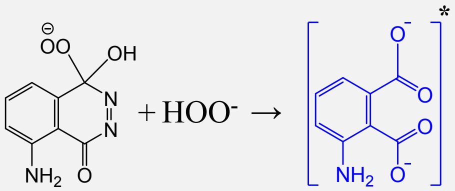
</td></tr>
<tr><td>10</td><td>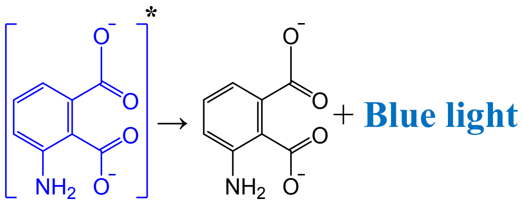
</td><td>5,14</td></tr>
<tr><td>11</td><td>Fe(III)-O- Fe(III) + $H^+$ → $Fe(III)^+$</td><td>16</td></tr>
</table>

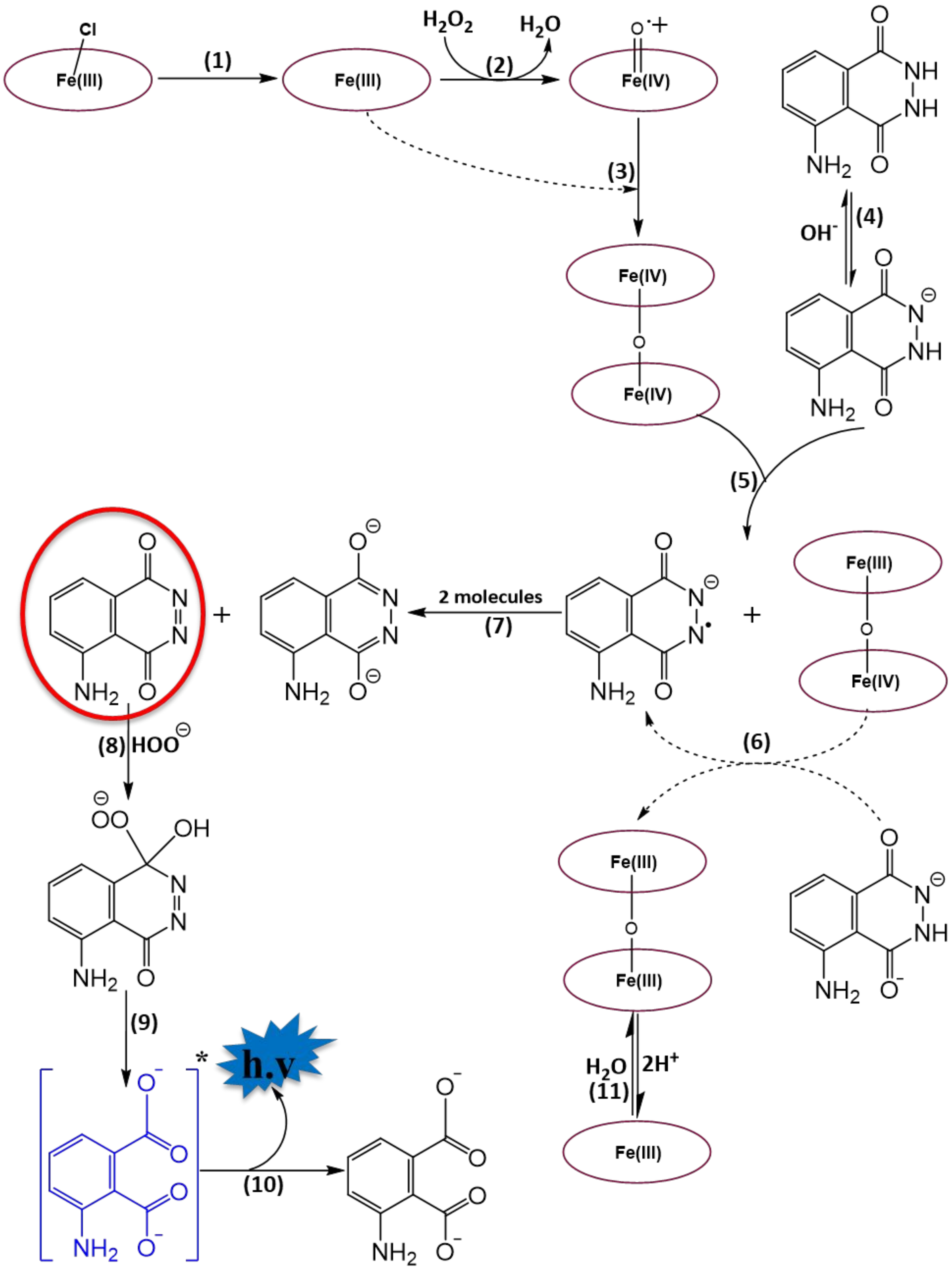


**Scheme S1.** Reaction mechanism postulated for the chemiluminescence (CL) oxidation of luminol in alkaline solution in the presence of hemin (Fe(III)).

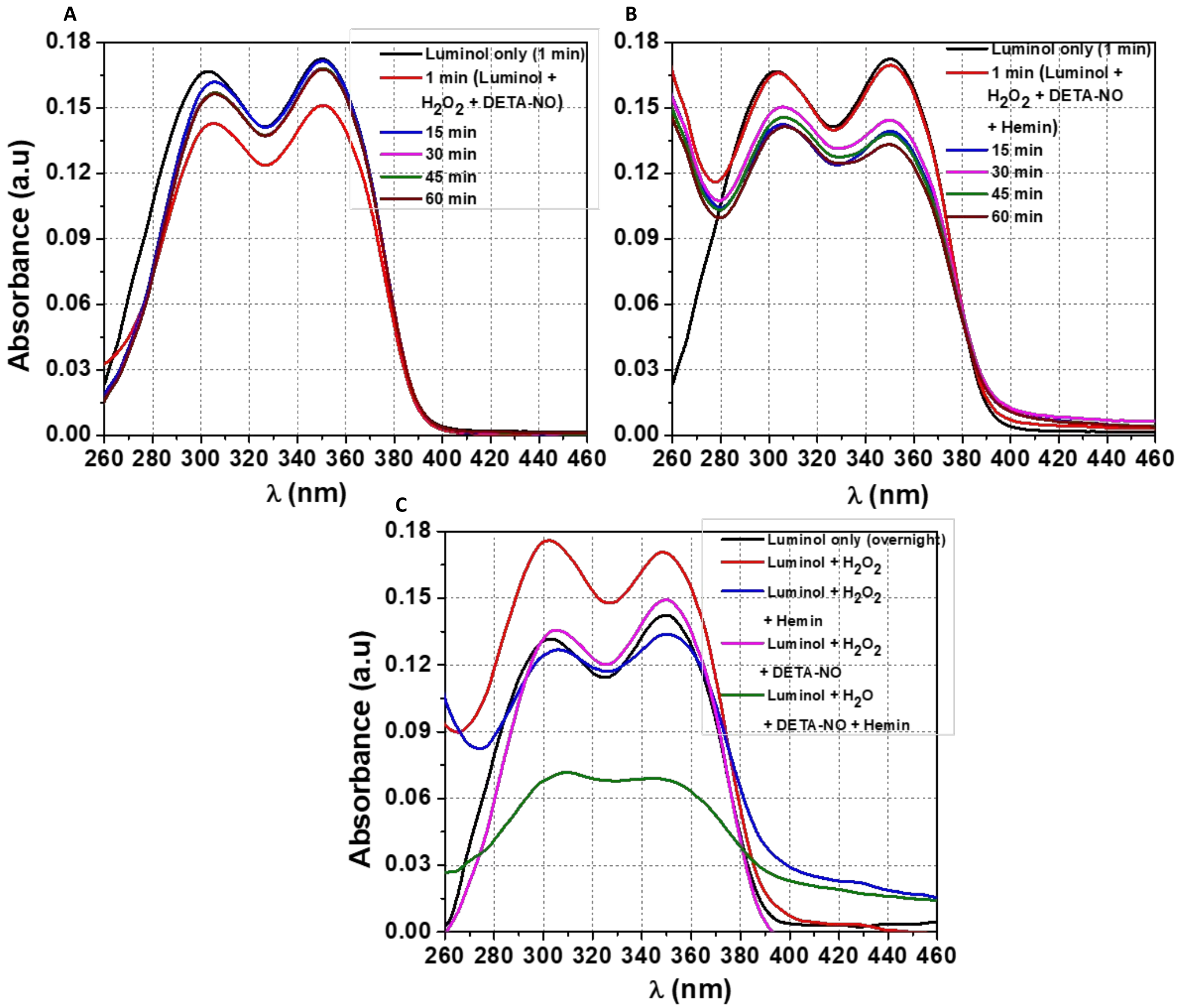


**Figure S2.** The overtime change in the UV-Vis spectra of 200 µM luminol **(black curve)** following mixing with 10 µM $H_2O_2$ and 60 µM DETA-NO **(A)** and with 10 µM $H_2O_2$, 1.6 µM hemin and 60 µM DETA-NO **(B)** for 1 **(red curve)**, 15 **(blue curve),** 30 **(magenta curve),** 45 **(olive curve)** and 60 minutes **(wine curve). (C)** The change in the UV-Vis spectra of 200 µM luminol **(black curve)** only and after mixing with 10 mM $H_2O_2$ only **(red curve)**, 10 mM $H_2O_2$, and 1.6 µM hemin **(blue curve),** 10 mM $H_2O_2$ and 60 µM DETA-NO **(magenta curve)**, and 10 mM $H_2O_2$, 1.6 µM hemin and 60 µM DETA-NO **(olive curve)** and overnight incubation. Results are presented as mean absorbance values, n = 3.

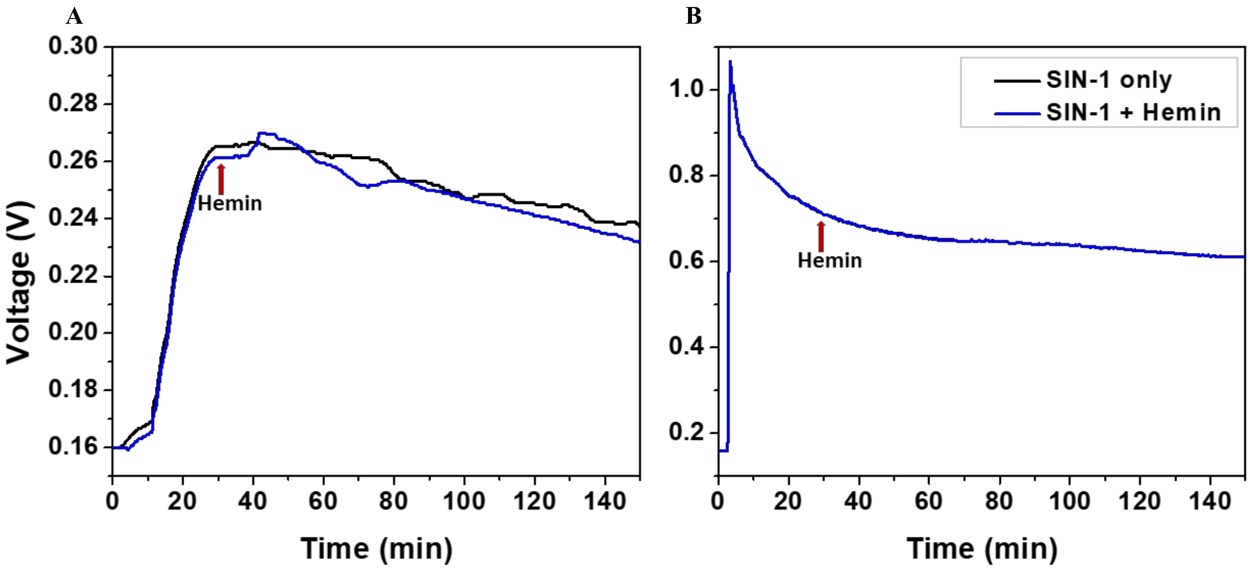


**Figure S3.** Temporal changes in the voltage signal in response to 1 mM SIN-1, prepared after thawing for 10 **(A)** and 30 min **(B)** and dissolving in phosphate buffer without **(black curve)** and with subsequent injection of 8 µM hemin **(blue curve)**. Results are presented as mean voltage values, n = 3.

**Table S2.** The main reactions involved in the hemin/$^{\bullet}NO/H_2O_2$/luminol-based CL reaction and light generation.

| NO. | REACTION | REFERENCES |
|---|---|---|
| 5 | $^{\bullet}NO + O_2^{\bullet -} \rightarrow ONOO^-$ | |
| 6 | 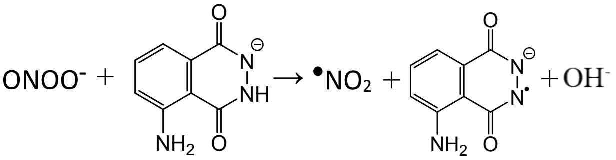 | 19,20 |
| 7 | $ONOO^- + Fe(IV){=}O^{\bullet +} \longrightarrow ONOO^{\bullet} + Fe(III)^+$ | 21-23 |
| 8 | $ONOO^- + H^+ \longleftrightarrow ONOOH \longrightarrow {}^{\bullet}NO_2 + HO^{\bullet} \longrightarrow NO_2^- + H^+ \longrightarrow NO_3^-$ | |
| 9 | $Fe(III)^+ + OH^- \longrightarrow Fe(III)\text{-}OH$ | 24 |
| 10 | $Fe(III)\text{-}OH + ONOO^- \longrightarrow Fe\text{-}[(OH)ONOO]^{2-}$ | |
| 11,12 | $Fe(III)^+ + {}^{\bullet}NO \longrightarrow Fe(III)\text{-}NO \longleftrightarrow [Fe(II)\text{-}NO]^+$ | 25 |
| 13 | $Fe(II)\text{-}NO^+ + H_2O \longrightarrow Fe(II) + NO_2^-$ | |
| 14,15 | $^{\bullet}NO + Fe(IV){=}O^{\bullet +} \longrightarrow Fe(IV){=}O + NO^+ \longrightarrow NO_2^-$ | 26,27 |
| 16 | $^{\bullet}NO + Fe(IV){=}O \longrightarrow Fe(III)^+ + NO^+$ | |
| 17 | $NO_2^- + Fe(IV){=}O \longrightarrow Fe(III)^+ + {}^{\bullet}NO_2$ | |

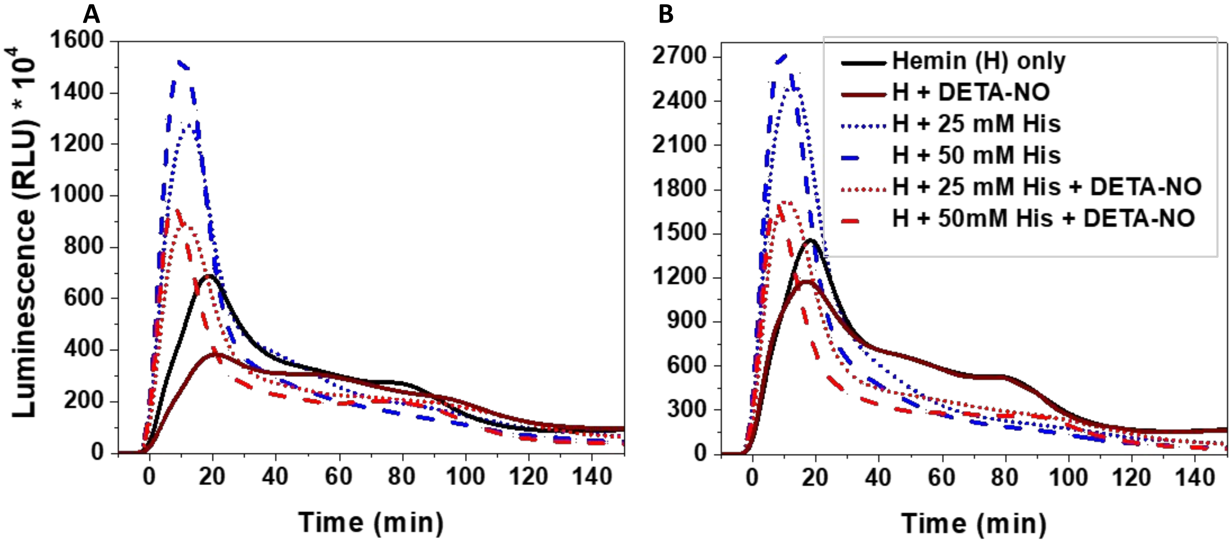


**Figure S4.** The $H_2O_2$/luminol-based luminescence kinetics in response to 4 **(A)** and 8 μM hemin **(B)** in the absence and presence of DETA-NO with and without L-histidine (His) in phosphate buffer; The concentrations: $H_2O_2$: 50 mM; luminol: 1 mM; DETA-NO: 300 μM in phosphate buffer (50 mM, pH 7.4). Results are presented as mean luminescence intensity values, n = 3.

**Computational studies**. Following dilution of hemin in aqueous solution, its hydroxide form is formed, with a possibility of oxidation in the presence of $H_2O_2$, or nitrosylation via the •NO. Moreover, a disproportionation equilibrium reaction was established between iron(IV)-oxo species (Fe(IV)=O), a high valent Fe-oxo species (HO-Fe(IV)=O), in the form of active radical cation, and hemin-hydroxide.[29] In addition, water was reported as a stabilizer for the Fe(IV) oxo porphyrin radical cations.[30,31] Hence, two forms of compound I were studied. **Tables S3-5** show the XYZ files in the case of hemin hydroxide, iron(IV)-oxo species, and (HO-Fe(IV)=O)-containing species, respectively, and **Figure S4** shows the geometry of these species in addition to that of nitrosylated hemin. In the geometry of the first oxidized form of hemin, the role of the axial ligand was neglected, with only the ferryl Fe(IV)=O moiety replacing the hydroxide group conjugated to central metal. Variations in the natural population analysis (NPA) charge of the central Fe in the case of Fe(IV)=O, HO-Fe(IV)=O, and $[Fe(II)\text{-}NO]^+$ were observed, which were also significantly different from those in the case of Fe(III)-OH and Fe(III)-Cl **(Table S6)**. These alterations in $[Fe(II)\text{-}NO]^+$ were explained before,[32] and relate mainly to the back donation of the unpaired electron from the N atom of •NO to a d orbital of Fe.[33,34] However, the charges on Fe of the oxidized forms were relatively higher, due to the oxidation reactions, resulting in less electron densities in the d-orbitals, responsible for the higher NPA charges. It is noteworthy to mention that these charges are calculated from the nuclear charge minus summed natural populations of all-natural atomic orbitals. These results were supported by the results of the natural electron configuration (NEC) **(Table S7).** In brief, the total occupation of all 3d orbitals was 6.531 and 6.556 in the case of Fe(IV)=O and HO-Fe(IV)=O, respectively, but reached 6.84 in $[Fe(II)\text{-}NO]^+$. Interestingly, the NPA charge of Fe in HO-Fe(IV)=O was less than that in the case of Fe(IV)=O, but with corresponding higher charges of the N-atoms of the pyrrole rings. These results indicate a significant flow of electrons from the orbitals of N-atoms in the case of HO-Fe(IV)=O to the central Fe, which was not significant in Fe(IV)=O, and this also explains the lower charge in the central Fe in the former case. However, a further investigation of these electron-donations was out of the scope of the recent work.

**Table S3.** XYZ-files of optimized geometry of hemin hydroxide ($FeC_{34}H_{33}N_4O_5$) (B3LYP, GenECP, S = 3/2). The basis sets: LANL2DZ for Fe atom; 6-31+G(d,p) for H atom**,** 6-31+G(d,p) for C, O and N atoms.

| | X | Y | Z |
|---|---|---|---|
| **Fe** | -0.96555 | -0.11406 | -0.02319 |
| **O** | 5.377421 | 1.682838 | 1.168248 |
| **O** | 3.486352 | 1.419208 | 2.270235 |
| **O** | 7.182078 | -2.46564 | -0.3833 |
| **O** | 7.110853 | -0.45666 | 0.5563 |
| **O** | -0.59842 | 0.014062 | 1.829721 |
| **N** | -2.08273 | -1.79298 | -0.05232 |
| **N** | 0.632903 | -1.21678 | -0.61939 |
| **N** | 0.070183 | 1.54969 | -0.47006 |
| **N** | -2.68201 | 0.972386 | -0.16091 |
| **C** | -3.41738 | -1.88517 | 0.264876 |
| **C** | 2.29727 | 0.569146 | -0.7132 |
| **C** | 1.435798 | 1.641172 | -0.53551 |
| **C** | 1.85111 | 3.02541 | - |

| | | | |
|---|---|---|---|
| | | 2 | 0.45293 |
| C | 0.703432 | 3.774063 | -0.38007 |
| C | -0.39646 | 2.837857 | -0.39718 |
| C | -1.7304 | 3.207996 | -0.3759 |
| C | -2.79822 | 2.328824 | -0.31873 |
| C | -4.19656 | 2.726501 | -0.26849 |
| C | -4.91071 | 1.583635 | 0.002804 |
| C | -3.95353 | 0.505584 | 0.058238 |
| C | -3.81039 | -3.2791 | 0.385264 |
| C | -4.29367 | -0.81588 | 0.302513 |
| C | -2.56789 | -5.51346 | 0.000478 |
| C | 2.520825 | -4.42015 | -1.15338 |
| C | 4.321337 | -1.7381 | -1.12353 |
| C | 5.011871 | -1.68278 | 0.262458 |
| C | 3.265557 | 3.522986 | -0.38904 |
| C | -2.70212 | -4.0228 | 0.052948 |
| C | 3.864526 | 3.52709 | 1.049639 |
| C | 0.55877 | 5.26278 | -0.29235 |
| C | -6.39201 | 1.429862 | 0.158015 |
| C | -1.63634 | -3.08586 | -0.19808 |
| C | -0.34396 | -3.45579 | -0.53589 |
| C | 0.711665 | -2.58214 | -0.72212 |
| C | 2.075705 | -3.0004 | -0.98118 |
| C | 2.83172 | -1.86172 | -0.99847 |
| C | 1.915697 | -0.75805 | -0.77635 |
| C | -5.12003 | -3.80536 | 0.765304 |

| | | | |
|---|---|---|---|
| C | -5.9494 | -3.30062 | 1.686408 |
| C | -4.74898 | 4.064938 | -0.46146 |
| C | -4.31468 | 4.999088 | -1.31636 |
| C | 4.308524 | 2.147721 | 1.498214 |
| C | 6.526471 | -1.58527 | 0.115016 |
| H | 4.730299 | -2.58473 | -1.67753 |
| H | 3.350228 | 0.785782 | -0.82217 |
| H | -1.9581 | 4.263525 | -0.36958 |
| H | -5.33531 | -1.03747 | 0.479676 |
| H | 2.357657 | -5.00561 | -0.24268 |
| H | 3.583765 | -4.47467 | -1.39037 |
| H | 1.975018 | -4.91593 | -1.96202 |
| H | 0.104751 | 5.567625 | 0.655834 |
| H | 1.524232 | 5.764488 | -0.37146 |
| H | -0.07758 | 5.650101 | -1.09369 |
| H | 4.583178 | -0.84339 | -1.69639 |
| H | 3.925895 | 2.945841 | -1.04138 |
| H | 3.303731 | 4.551059 | -0.75472 |
| H | -0.13956 | -4.51277 | -0.63557 |
| H | -5.41997 | -4.72147 | 0.262659 |
| H | -6.89709 | -3.77986 | 1.899046 |
| H | -5.69967 | -2.42439 | 2.272281 |
| H | -4.8092 | 5.960388 | -1.3825 |
| H | -5.62556 | 4.298237 | 0.137843 |
| H | -3.48569 | 4.82575 | -1.99156 |
| H | 2.60652 | 1.81342 | 2.35515 |

| | | | |
|---|---|---|---|
| | 8 | 2 | |
| **H** | 6.462889 | 0.193876 | 0.88665 |
| **H** | 4.606921 | -0.86242 | 0.856547 |
| **H** | 4.802517 | -2.61187 | 0.798322 |
| **H** | 4.764351 | 4.14452 | 1.055083 |
| **H** | 3.144046 | 3.961793 | 1.747043 |
| **H** | -2.16576 | -5.84632 | -0.96096 |
| **H** | -1.89451 | -5.88165 | 0.781411 |
| **H** | -3.53087 | -6.00353 | 0.146065 |
| **H** | -6.89117 | 2.399066 | 0.178237 |
| **H** | -6.64628 | 0.904247 | 1.083155 |
| **H** | -6.82326 | 0.858405 | -0.67041 |
| **H** | -0.86241 | -0.72544 | 2.388389 |

**Table S4.** XYZ-files of optimized geometry of oxo iron(IV) porphyrin p-cation radical species **1** (Fe(IV)=O) ($[FeC_{34}H_{32}N_4O_5]^+$) (B3LYP, GenECP, S = 3/2). The basis sets: LANL2DZ for Fe atom; 6-31+G(d,p) for H atom**,** 6-31+G(d,p) for C, O and N atoms.

| | X | Y | Z |
|---|---|---|---|
| **Fe** | -1.03166 | -0.112576 | -0.03423 |
| **O** | 5.081694 | 1.288633 | 0.634727 |
| **O** | 5.246365 | 2.377971 | 2.541287 |
| **O** | 6.95697 | -3.001935 | -0.08799 |
| **O** | 6.961329 | -0.856726 | 0.491091 |
| **O** | -0.79965 | -0.103062 | 1.553722 |
| **N** | -2.2623 | -1.695701 | -0.0878 |
| **N** | 0.482671 | -1.305261 | -0.5639 |
| **N** | 0.128783 | 1.469186 | -0.44416 |
| **N** | - | 1.080532 | - |

| | | | |
|---|---|---|---|
| | 2.63995 | | 0.12324 |
| C | -3.58617 | -1.701603 | 0.275646 |
| C | 2.28899 | 0.347739 | -0.62277 |
| C | 1.503809 | 1.474303 | -0.52326 |
| C | 2.014836 | 2.848379 | -0.5465 |
| C | 0.925025 | 3.659338 | -0.48629 |
| C | -0.24263 | 2.780313 | -0.41661 |
| C | -1.5479 | 3.249683 | -0.35885 |
| C | -2.66322 | 2.446989 | -0.25532 |
| C | -4.04011 | 2.953805 | -0.14478 |
| C | -4.81749 | 1.873714 | 0.157497 |
| C | -3.9329 | 0.718348 | 0.155101 |
| C | -4.08558 | -3.08154 | 0.38968 |
| C | -4.37488 | -0.577787 | 0.384641 |
| C | -3.01269 | -5.380546 | -0.07585 |
| C | 2.150155 | -4.646883 | -1.06791 |
| C | 4.144024 | -2.095488 | -0.97062 |
| C | 4.82328 | -2.047641 | 0.422063 |
| C | 3.453458 | 3.273363 | -0.67068 |
| C | -3.0525 | -3.889013 | 0.012753 |
| C | 4.182393 | 3.525134 | 0.669084 |
| C | 0.854525 | 5.152809 | -0.50358 |
| C | -6.29282 | 1.816787 | 0.39061 |
| C | -1.91771 | -3.01173 | -0.23805 |
| C | -0.64623 | -3.476612 | -0.54875 |
| C | 0.468752 | -2.678804 | -0.67139 |

| | | | |
|---|---|---|---|
| **C** | 1.819039 | -3.200739 | -0.88821 |
| **C** | 2.650567 | -2.126765 | -0.86673 |
| **C** | 1.798914 | -0.951709 | -0.66823 |
| **C** | -5.42177 | -3.503805 | 0.799927 |
| **C** | -6.15756 | -2.956307 | 1.774525 |
| **C** | -4.48735 | 4.333188 | -0.31267 |
| **C** | -4.0249 | 5.213036 | -1.20899 |
| **C** | 4.852414 | 2.296945 | 1.262704 |
| **C** | 6.342522 | -2.029104 | 0.259663 |
| **H** | 4.502406 | -2.980641 | -1.4981 |
| **H** | 3.357622 | 0.484332 | -0.68 |
| **H** | -1.70062 | 4.317916 | -0.36051 |
| **H** | -5.42131 | -0.718883 | 0.606791 |
| **H** | 1.890912 | -5.227724 | -0.17714 |
| **H** | 3.21435 | -4.788296 | -1.25291 |
| **H** | 1.604568 | -5.078803 | -1.91223 |
| **H** | 0.409331 | 5.541361 | 0.417661 |
| **H** | 1.84385 | 5.598472 | -0.60506 |
| **H** | 0.243688 | 5.513486 | -1.33652 |
| **H** | 4.463485 | -1.233793 | -1.56397 |
| **H** | 4.026457 | 2.542712 | -1.24359 |
| **H** | 3.484355 | 4.2009 | -1.24536 |
| **H** | -0.51523 | -4.543757 | -0.65505 |
| **H** | -5.81641 | -4.375684 | 0.286512 |
| **H** | -7.12979 | -3.361316 | 2.024797 |
| **H** | - | -2.12802 | 2.37652 |

| | | | |
|---|---|---|---|
| | 5.80356 | | 3 |
| **H** | -4.4448 | 6.208879 | -1.2709 |
| **H** | -5.30688 | 4.639951 | 0.330518 |
| **H** | -3.25592 | 4.96156 | -1.92942 |
| **H** | 5.018836 | 3.227544 | 2.942561 |
| **H** | 6.331608 | -0.149223 | 0.717484 |
| **H** | 4.465929 | -1.188503 | 0.992248 |
| **H** | 4.569058 | -2.952758 | 0.976761 |
| **H** | 4.990816 | 4.248653 | 0.506821 |
| **H** | 3.507373 | 3.974181 | 1.403983 |
| **H** | -2.68709 | -5.714085 | -1.06549 |
| **H** | -2.3198 | -5.802555 | 0.65904 |
| **H** | -3.99317 | -5.814028 | 0.117181 |
| **H** | -6.73788 | 2.810224 | 0.352868 |
| **H** | -6.52588 | 1.381603 | 1.366936 |
| **H** | -6.79117 | 1.206153 | -0.3687 |

**Table S5.** XYZ-files of optimized geometry of oxo iron(IV) porphyrin p-cation radical species **2** (HO-Fe(IV)=O) ($[FeC_{34}H_{33}N_4O_6]^+$) (B3LYP, GenECP, S = 3/2). The basis sets: LANL2DZ for Fe atom; 6-31+G(d,p) for H atom**,** 6-31+G(d,p) for C, O and N atoms.

| | X | Y | Z |
|---|---|---|---|
| **Fe** | 1.058662 | -0.08272 | 0.010974 |
| **O** | -5.48104 | 1.290217 | 0.105333 |
| **O** | -6.25402 | 3.340996 | -0.14776 |
| **O** | -7.07789 | -3.23031 | -0.43345 |
| **O** | -7.15247 | -1.08949 | 0.143379 |
| **O** | 0.966827 | -0.05495 | -1.65041 |
| **O** | 1.101609 | -0.12985 | 1.936124 |

| | | | |
|---|---|---|---|
| N | 2.249015 | -1.70259 | -0.01497 |
| N | -0.54533 | -1.26131 | 0.228071 |
| N | -0.14041 | 1.539731 | 0.028702 |
| N | 2.698936 | 1.085122 | 0.028131 |
| C | 3.584991 | -1.73337 | -0.34565 |
| C | -2.28703 | 0.415062 | -0.00286 |
| C | -1.46083 | 1.538853 | -0.1567 |
| C | -1.93446 | 2.936021 | -0.32419 |
| C | -0.77017 | 3.758054 | -0.04725 |
| C | 0.288167 | 2.877 | 0.103054 |
| C | 1.640697 | 3.260543 | 0.287815 |
| C | 2.735961 | 2.446973 | 0.265435 |
| C | 4.12803 | 2.914379 | 0.276338 |
| C | 4.891349 | 1.830136 | -0.06154 |
| C | 3.969388 | 0.708801 | -0.18232 |
| C | 4.059761 | -3.12469 | -0.40334 |
| C | 4.383807 | -0.62106 | -0.42818 |
| C | 2.934049 | -5.38377 | 0.135461 |
| C | -2.25244 | -4.48357 | 1.129351 |
| C | -4.21093 | -1.93193 | 0.782616 |
| C | -5.00041 | -2.03556 | -0.5472 |
| C | -3.10432 | 3.42744 | -0.7989 |
| C | 3.004058 | -3.89756 | -0.01062 |
| C | -4.25027 | 2.676433 | -1.41905 |
| C | -0.74535 | 5.248799 | -0.04076 |
| C | 6.37628 | 1.74238 | - |

| | | | |
|---|---|---|---|
| | 8 | 9 | 0.21943 |
| C | 1.881383 | -2.98465 | 0.188088 |
| C | 0.579032 | -3.4024 | 0.494513 |
| C | -0.54331 | -2.59584 | 0.511494 |
| C | -1.90638 | -3.07562 | 0.764877 |
| C | -2.72324 | -2.00195 | 0.604819 |
| C | -1.84778 | -0.87378 | 0.255326 |
| C | 5.394962 | -3.58 | -0.78092 |
| C | 6.148257 | -3.06779 | -1.76103 |
| C | 4.596146 | 4.265174 | 0.571554 |
| C | 4.098074 | 5.077108 | 1.511924 |
| C | -5.38197 | 2.368194 | -0.42517 |
| C | -6.49841 | -2.18863 | -0.29217 |
| H | -4.52472 | -2.7497 | 1.434966 |
| H | -3.35458 | 0.575089 | 0.012371 |
| H | 1.831553 | 4.319613 | 0.383356 |
| H | 5.436517 | -0.78132 | -0.61262 |
| H | -1.97647 | -5.17996 | 0.331184 |
| H | -3.32183 | -4.59802 | 1.302714 |
| H | -1.73154 | -4.79957 | 2.038125 |
| H | -0.85146 | 5.65026 | -1.0547 |
| H | -1.56457 | 5.652011 | 0.560909 |
| H | 0.184023 | 5.641629 | 0.368585 |
| H | -4.47816 | -1.00856 | 1.30336 |
| H | -3.19868 | 4.509419 | -0.81151 |
| H | 0.434262 | -4.45641 | 0.689669 |

| | | | |
|---|---|---|---|
| H | 5.774461 | -4.4378 | -0.23349 |
| H | 7.122049 | -3.48439 | -1.98469 |
| H | 5.806612 | -2.2567 | -2.39334 |
| H | 4.522194 | 6.059643 | 1.675382 |
| H | 5.450575 | 4.607284 | -0.0051 |
| H | 3.293664 | 4.775043 | 2.172493 |
| H | -6.10913 | 4.137268 | -0.6759 |
| H | -6.58154 | -0.30663 | 0.198989 |
| H | -4.80996 | -1.15733 | -1.17096 |
| H | -4.68413 | -2.91922 | -1.10055 |
| H | -4.64759 | 3.27537 | -2.245 |
| H | -3.9231 | 1.723256 | -1.82954 |
| H | 2.589606 | -5.67271 | 1.132901 |
| H | 2.244087 | -5.82276 | -0.59229 |
| H | 3.908975 | -5.84225 | -0.02601 |
| H | 6.842649 | 2.720849 | -0.10955 |
| H | 6.652386 | 1.349912 | -1.20266 |
| H | 6.820359 | 1.084472 | 0.534272 |
| H | 1.996043 | -0.15192 | 2.29554 |

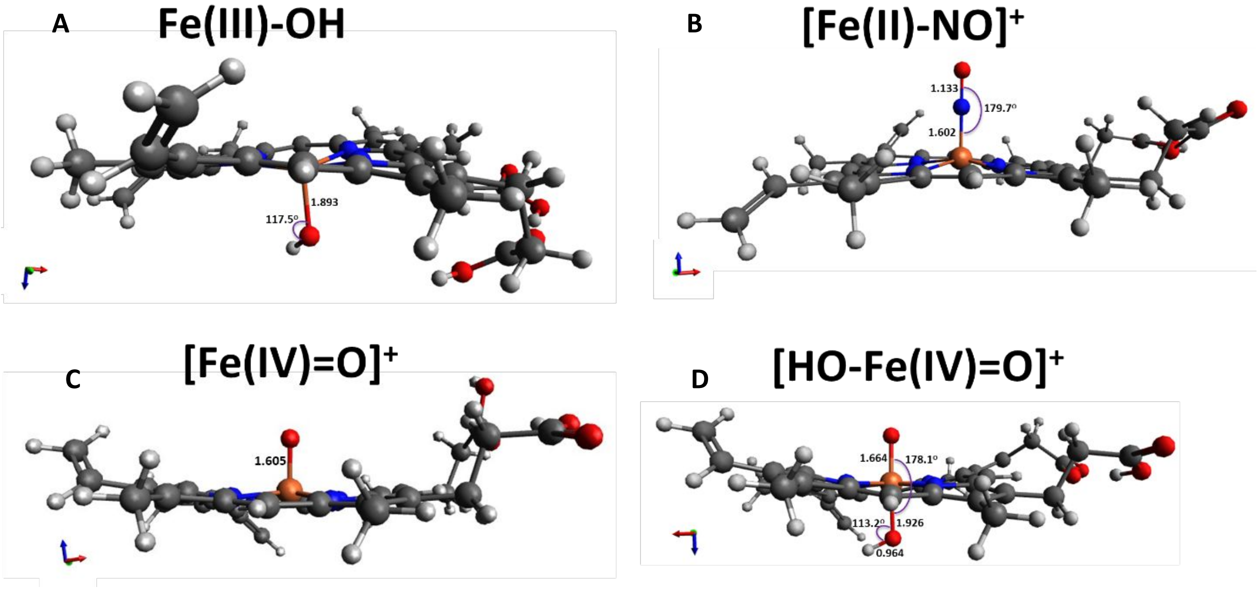


**Figure S5.** The optimized molecular geometries of hemin hydroxide **(A)**, nitrosylated hemin **(B)**, iron(IV)-oxo species **(C)** and (HO-Fe(IV)=O)-containing species **(D)**.

**Table S6.** Comparison of the distribution of the NPA charges among the optimized geometries of hemin (Fe(III)-Cl) (S = 3/2), hemin hydroxide (Fe(III)-OH) (S = 3/2), oxo iron(IV) porphyrin p-cation radical species **1** (Fe(IV)=O) (S = 3/2) and **2** (HO-Fe(IV)=O) (S = 3/2) and hemin in complexation with •NO based on the $[Fe(II)\text{-}NO]^+$ (S = 0) electronic state at the level of DFT/B3LYP/Gen ECP via Polarizable Continuum Model (PCM) using the integral equation formalism variant (IEFPCM). The basis sets: LANL2DZ for the Fe atom, 6-31+G(d,p) for H atom**,** 6-31+G(d,p) for C, O, N and Cl atoms**.** All energies are in eV.

| | **Fe(III)** | **Fe(III)-OH** | **Fe(IV)=O** | **HO-Fe(IV)=O** | **$[Fe(II)\text{-}NO]^+$** |
|---|---|---|---|---|---|
| **Fe** | 0.862 | 0.96311 | 0.68033 | 0.43446 | 0.272 |
| **N*** | - | - | - | - | 0.591 |
| **O*** | - | - | - | - | -0.066 |
| **NO*** | - | - | - | - | 0.525 |
| **O’** | | | -0.35442 | -0.39820 | - |
| **O** | - | -0.95297 | - | -0.89071 | - |
| **H** | - | 0.46293 | - | 0.45438 | - |
| **OH** | - | -0.49004 | - | -0.43633 | - |
| **N1** | -0.492 | -0.49136 | -0.45327 | -0.34493 | -0.407 |
| **N2** | -0.496 | -0.49172 | -0.45679 | -0.32941 | -0.408 |
| **N3** | -0.493 | -0.48456 | -0.45177 | -0.27697 | -0.409 |

| | | | | | |
|---|---|---|---|---|---|
| **N4** | -0.494 | -0.48827 | -0.45445 | -0.34830 | -0.405 |
| **Cl** | -0.525 | - | - | - | - |

**Table S7.** The NEC in the iron atom of hemin (Fe(III)-Cl) (S = 3/2), hemin hydroxide (Fe(III)-OH) (S = 3/2), oxo iron(IV) porphyrin p-cation radical species **1** ($[Fe(IV)=O]^+$) (S = 3/2) and **2** ($[HO\text{-}Fe(IV)=O]^+$) (S = 3/2) and hemin in complexation with •NO based on the $[Fe(II)\text{-}NO]^+$ (S = 0) electronic state with the occupation of the orbitals in the 3d level.

| | **NEC** | **Occupation of 3d orbitals** |
|---|---|---|
| **Fe(III)-Cl** | $4S^{0.27}3d^{6.29}4p^{0.53}4d^{0.03}$ | $(3d_{xy})^{1.136}(3d_{xz})^{1.175}(3d_{yz})^{1.114}(3d_{x2-y2})^{1.628}(3d_{z2})^{1.234}$ |
| **Fe(III)-OH** | $4S^{0.25}3d^{6.26}4p^{0.49}4d^{0.03}$ | $(3d_{xy})^{0.984}(3d_{xz})^{1.173}(3d_{yz})^{1.098}(3d_{x2-y2})^{1.756}(3d_{z2})^{1.247}$ |
| **$[Fe(IV)=O]^+$** | $4S^{0.25}3d^{6.53}4p^{0.53}4d^{0.03}$ | $(3d_{xy})^{0.984}(3d_{xz})^{1.173}(3d_{yz})^{1.098}(3d_{x2-y2})^{1.756}(3d_{z2})^{1.247}$ |
| **$[HO\text{-}Fe(IV)=O)]^+$** | $4S^{0.26}3d^{6.62}4p^{0.62}4d^{0.03}$ | $(3d_{xy})^{0.905}(3d_{xz})^{1.41}(3d_{yz})^{1.437}(3d_{x2-y2})^{1.853}(3d_{z2})^{0.951}$ |
| **$[Fe(II)\text{-}NO]^+$** | $4S^{0.27}3d^{6.84}4p^{0.60}4d^{0.03}$ | $(3d_{xy})^{0.977}(3d_{xz})^{1.555}(3d_{yz})^{1.554}(3d_{x2-y2})^{1.859}(3d_{z2})^{0.900}$ |

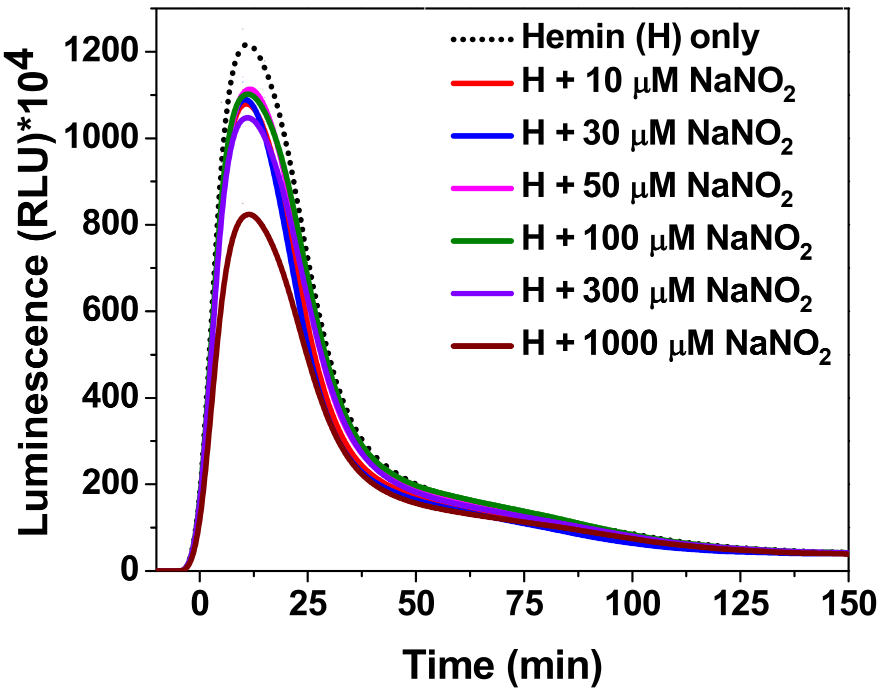


**Figure S6** The $H_2O_2$/luminol-based luminescence kinetics, measured at 425 nm, in response to 8 μM hemin mixed with different concentrations of $NaNO_2$. The concentrations: $H_2O_2$: 50 mM; luminol: 1 mM in phosphate buffer (50 mM, pH 7.4). Results are presented as mean luminescence intensity values, n = 3.

**The CL reaction and luminescence generation are affected by solution pH and metal ions.** The importance of luminol and $H_2O_2$ as main components of the CL reactions was first examined in PB in the presence and absence of hemin. Both reagents were essential for the luminescence generation, and their absence did not induce any CL reaction in the presence of hemin **(Figure S7A)**. Furthermore, the freshness of luminol played a role, where one-week-old luminol prepared in DMSO/buffer mixture was less efficient in enhancing the hemin/$H_2O_2$/luminol-CL reaction, compared to that prepared freshly before the main experiments **(Figure S7B)**. Moreover, as explained in **Schematic S1,** the rate of CL reaction is controlled by the pH of the solution and $H_2O_2$ concentration. Hence, at pH 7.4, the luminescence kinetics were enhanced with the increase in $H_2O_2$ concentration, up to 50 mM, followed by a decay at 100 mM **(Figure S7C)**. Similar behavior was observed previously.[35] This later decay in light yield was reported can be due to suicide inactivation, where certain high $H_2O_2$ concentrations at pH $\leq$ 9 were reported to cause disintegration of the porphyrin ring with liberation of the central iron.[36,37] Hence, the liberated iron ions, owing to their efficiency to enhance the CL reaction but with lower affinity than hemin itself, cause a transition from the highly efficient flash to less efficient glow luminescence kinetics.[35] These changes are responsible for the initial decrease in luminescence intensity at 100 mM $H_2O_2$. However, the slight increase in kinetics observed at 50 and 100 mM $H_2O_2$ refers to further activation of the CL reaction over a prolonged time of measurement. This is mainly due to the recycling of the reduced hemin-dimers, formed in Reactions **6** and **11** (**Scheme S1)** by excess $H_2O_2$, which can fuel the CL reaction further. An example of this less efficient glow luminescence due to PPIX, $FeCl_2$, and $FeCl_3$ is shown in **S7D.** For comparison, when the CL reaction was performed at pH 10.5 using carbonate buffer, a generally expected enhancement in light yield was observed **(Figure S7E)**, as explained before**.** Moreover, the enhanced effects of the same $H_2O_2$ concentration with the increase in pH are due to its activated degradation, particularly as its pKa value is 11.7.[7,13] This leads to the formation of peroxide anion, reported to have higher oxidizing effects than $H_2O_2$ itself, causing an increase in the light yield (**Schematic S1, Reaction 8,9)**. According to these results, all the CL reactions were performed using 50 mM $H_2O_2$ in PB and cell culture medium, with a pH of around 7.4.

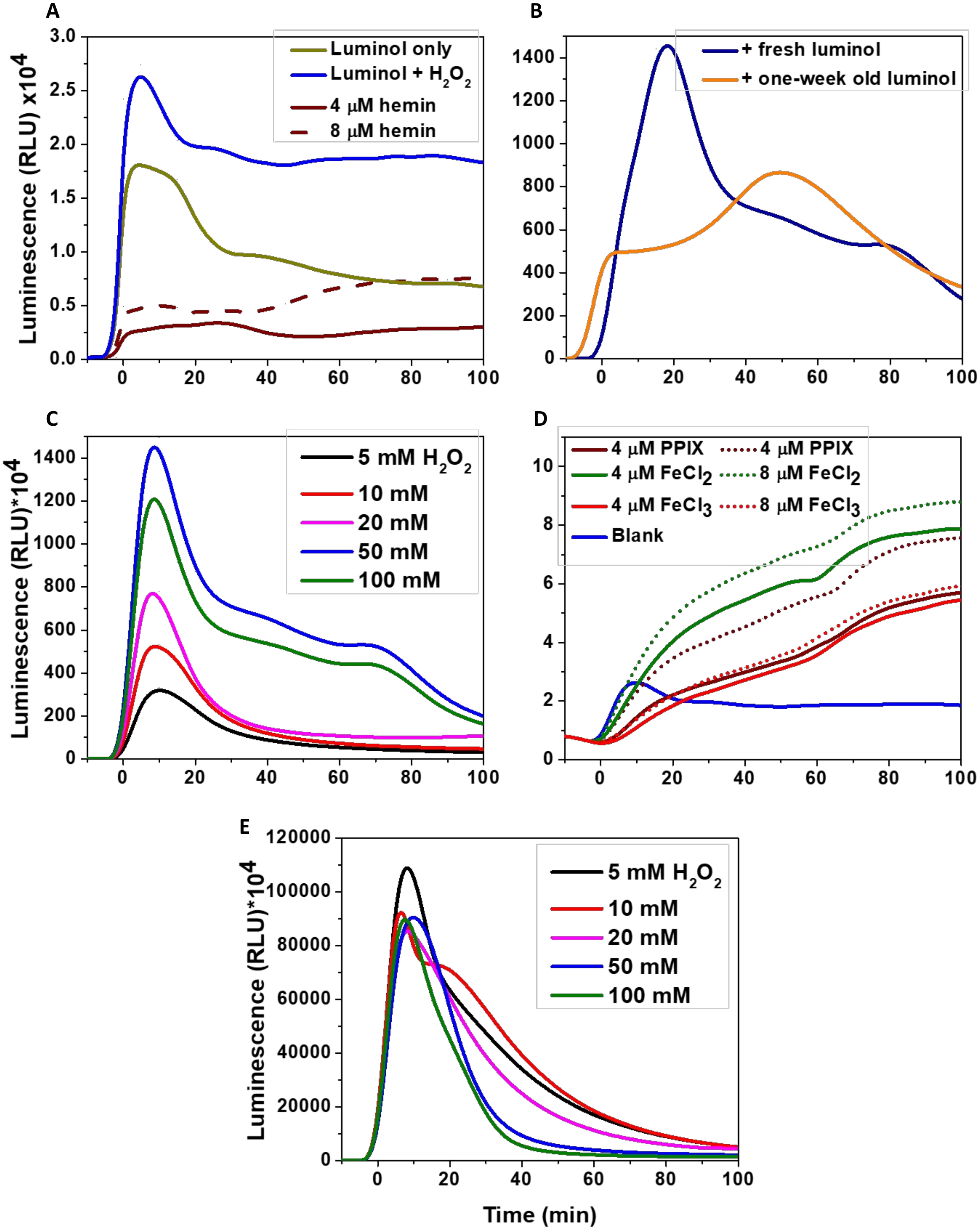


**Figure S7** The luminescence kinetics in phosphate buffer (50 mM, pH 7.4), measured at 425 nm, in response to: **(A)** 1 mM luminol only, mixture of luminol and 50 mM $H_2O_2$ only, or hemin only, **(B)** mixture of 50 mM $H_2O_2$ and 1 mM fresh or one-week old luminol, **(C)** mixture of 1 mM fresh luminol and different concentrations of $H_2O_2$, **(D)** mixture of 1 mM

luminol and 50 mM $H_2O_2$ in combination with different concentrations of protoporphyrin IX (PPIX), $FeCl_2$ or $FeCl_3$. **(E)** The luminescence kinetics in carbonate buffer (50 mM, pH 10.5) in response to a mixture of 1 mM fresh luminol and different concentrations of $H_2O_2$. Results are presented as mean luminescence intensity values, n = 3.

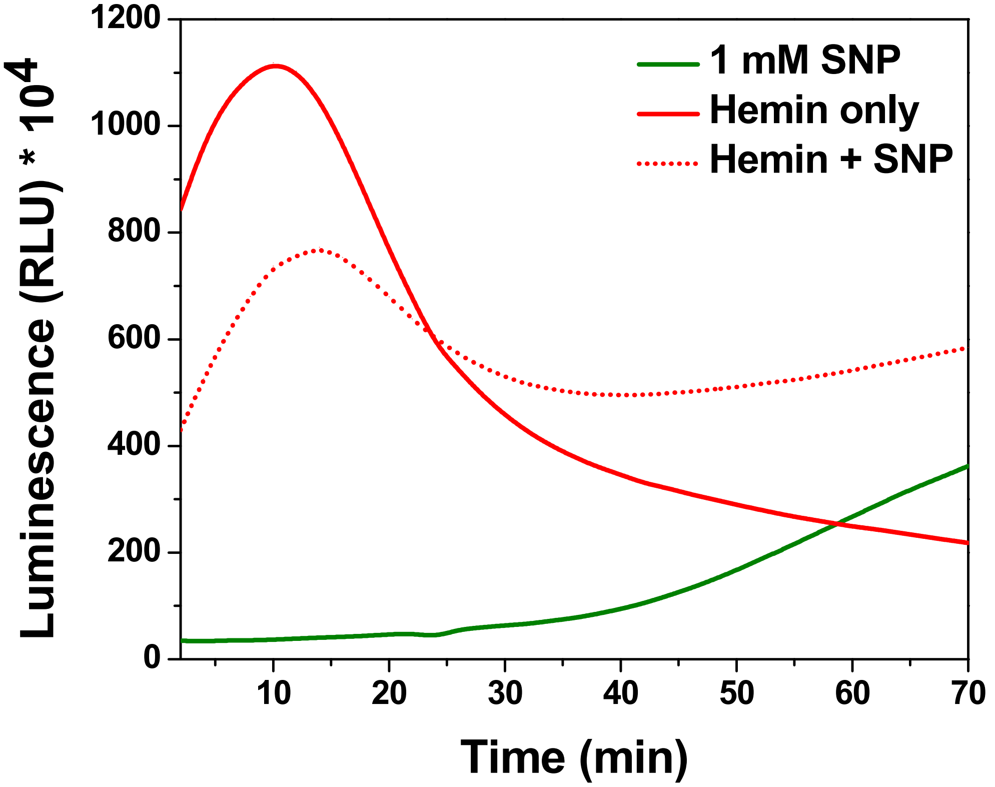


**Figure S8** The $H_2O_2$/luminol-based luminescence kinetics, measured at 425 nm, in response to SNP only, 8 μM hemin only, or after mixing of hemin with the CL reagents, followed by addition of SNP in phosphate buffer (50 mM, pH 7.4). Results are presented as mean luminescence intensity values, n = 3.

## REFERENCES


(1) Dounce, A. L.; Sichak, S. P. *Free Radic. Biol. Med.* **1988**, *5* (2), 89–93.
(2) Nakamura, M.; Nakamura, S. *Free Radic. Biol. Med.* **1998**, *24* (4), 537–544.
(3) Cormier, M. J.; Prichard, P. M. *J. Biol. Chem.* **1968**, *243* (18), 4706–4714.
(4) Davies, D. M.; Jones, P.; Mantle, D. *Biochem. J.* **1976**, *157* (1), 247–253.
(5) Bastos, E. L.; Ciscato, L. F. M. L.; Bartoloni, F. H.; Catalani, L. H.; Baader, W. J. *Luminescence* **2007**, *22* (2), 113–125.
(6) Erdey, L.; Buzás, I.; Vigh, K. *Talanta* **1966**, *13* (3), 463–469.
(7) Haapakka, K. E.; Kankare, J. J. *Anal. Chim. Acta* **1982**, *138* (C), 263–275.
(8) Schiller, J.; Arnhold, J.; Schwinn, J.; Sprinz, H.; Brede, O.; Arnold, K. *Free Radic. Res.* **1999**, *30* (1), 45–57.
(9) Jones, P.; Scowen, N. R. *Photochem. Photobiol.* **1987**, *45* (2), 283–289.
(10) Candy, T. E. G.; Hodgson, M.; Jones, P. *J. Chem. Soc. Perkin Trans. 2* **1990**, No. 8, 1385.
(11) Merényi, G.; Lind, J.; Eriksen, T. E. *J. Biolumin. Chemilumin.* **1990**, *5* (1), 53–56.
(12) Merenyi, G.; Lind, J.; Erikson, T. E. *J. Am. Chem. Soc.* **1986**, *108* (24), 7716–7726.
(13) Merenyi, G.; Lind, J. S. A Mechanistic Study. *J. Am. Chem. Soc.* **1980**, *102* (18), 5830–5835.
(14) Baader, W. J.; Stevani, C. V.; Bastos, E. L. *ChemInform* **2007**, *38* (1).
(15) Bastos, E. L.; Romoff, P.; Eckert, C. R.; Baader, W. J. *J. Agric. Food Chem.* **2003**, *51* (25), 7481–7488.
(16) De Villiers, K. A.; Kaschula, C. H.; Egan, T. J.; Marques, H. M. *J. Biol. Inorg. Chem.* **2007**, *12* (1), 101–117.
(17) Golnak, R.; Xiao, J.; Atak, K.; Stevens, J. S.; Gainar, A.; Schroeder, S. L. M.; Aziz, E. F. *Phys. Chem. Chem. Phys.* **2015**, *17* (43), 29000–29006.
(18) Tan, X.; Song, Z.; Chen, D.; Wang, Z. *Spectrochim. Acta Part A Mol. Biomol. Spectrosc.* **2011**, *79* (1), 232–235.
(19) Radi, R.; Beckman, J. S.; Bush, K. M.; Freeman, B. A. *J. Biol. Chem.* **1991**, *266* (7), 4244–4250.
(20) Radi, R.; Beckman, J. S.; Bush, K. M.; Freeman, B. A. *Arch. Biochem. Biophys.* **1991**, *288* (2), 481–487.
(21) Ferrer-Sueta, G.; Batinić-Haberle, I.; Spasojević, I.; Fridovich, I.; Radi, R. *Chem. Res. Toxicol.* **1999**, *12* (5), 442–449.
(22) Jensen, M. P.; Riley, D. P. *Inorg. Chem.* **2002**, *41* (18), 4788–4797.
(23) Peteu, S. F.; Bose, T.; Bayachou, M. *Anal. Chim. Acta* **2013**, *780*, 81–88.
(24) Fotiou, S.; Fotiou, D.; Deliconstantinos, G. *In Vivo* **2009**, *23* (2), 281–286.
(25) Alsharabasy, A. M.; Glynn, S.; Farràs, P.; Pandit, A. *Nitric Oxide* **2022**, *124*, 49–67.
(27) Abu-Soud, H. M.; Hazen, S. L. *Free Radic. Biol. Med.* **1999**, *27*, S71.
(28) Abu-Soud, H.; Khassawneh, M. Y.; Sohn, J. T.; Murray, P.; Haxhiu, M. A.; Hazen, S. L. *Biochemistry* **2001**, *40* (39), 11866–11875.
(29) Pan, Z.; Newcomb, M. *Inorg. Chem.* **2007**, *46* (16), 6767–6774.
(30) Pan, Z.; Zhang, R.; Newcomb, M. *J. Inorg. Biochem.* **2006**, *100* (4), 524–532.
(31) Bell, S. E. J.; Cooke, P. R.; Inchley, P.; Leanord, D. R.; Smith, J. R. L.; Robbins, A. *J. Chem. Soc. Perkin Trans. 2* **1991**, No. 4, 549.
(32) Pandit, A.; Alsharabasy, A.; Warneke, J.; Warneke, Z.; Glynn, S.; Farràs, P. *ChemRxiv* **2022**. (33) Addison, A. W.; Stephanos, J. J. *Biochemistry* **1986**, *25* (14), 4104–4113.
(34) Praneeth, V. K. K.; Paulat, F.; Berto, T. C.; George, S. D.; Näther, C.; Sulok, C. D.; Lehnert, N. *J. Am. Chem. Soc.* **2008**, *130* (46), 15288–15303.
(35) Plieth, C. *ACS Omega* **2019**, *4* (2), 3268–3279.
(36) Arnao, M. B.; Acosta, M.; del Rio, J. A.; García-Cánovas, F. *Biochim. Biophys. Acta - Protein Struct. Mol. Enzymol.* **1990**, *1038* (1), 85–89.
(37) Valderrama, B.; Ayala, M.; Vazquez-Duhalt, R. *Chem. Biol.* **2002**, *9* (5), 555–565.
